# Brain injury after single fetal demise in monochorionic twin pregnancies: an international multicentre retrospective cohort study

**DOI:** 10.1016/j.eclinm.2026.104290

**Published:** 2026-10-28

**Authors:** Mathies Rondagh, Liesbeth Lewi, Michael Aertsen, Francesca Russo, Mariano Lanna, Daniela Casati, Andrea Righini, Shiri Shinar, Elka Miller, Laurence S. Carmant, Asma Khalil, Sara Sorrenti, Doaa Mohammed, Lotta Herling, Kajsa Bohlin, Yoav Yinon, Daphna Amitai Komem, Daniel Sanin-Ramirez, Paola Roca, Cesar H. Meller, Lucas Otaño, Javier U. Ortiz, Isabella Fabietti, Lotte C.M. Zwinkels, Femke Slaghekke, Monique C. Haak, Jeanine M.M. van Klink, Linda S. de Vries, Sophie G. Groene, Sylke J. Steggerda, Enrico Lopriore, the CITRUS Study Group, Ana Alarcón, Mar Bennasar, Giana Izzo, Daniela Longo, Marcelo Pietrani, Borna Poljak, Sara Savelli, Irene Spektor, Federica Torracca

**Affiliations:** a Division of Neonatology, Department of Paediatrics, Willem-Alexander Children’s Hospital, Leiden University Medical Centre, the Netherlands; b Department of Obstetrics and Gynaecology, University Hospitals Leuven, Leuven, Belgium; c Department of Development and Regeneration, KU Leuven, Leuven, Belgium; d Department of Radiology, University Hospitals Leuven, Leuven, Belgium; e Department of Imaging and Pathology, KU Leuven, Leuven, Belgium; f Fetal Therapy Unit ‘U. Nicolini’, ‘Vittore Buzzi’ Children’s Hospital, University of Milan, Milan, Italy; g Paediatric Radiology and Neuroradiology Unit, Vittore Buzzi Children’s Hospital, Milan, Italy; h Division of Maternal Fetal Medicine, Department of Obstetrics and Gynaecology, Ontario Fetal Centre, Mount Sinai Hospital, University of Toronto, Toronto, Ontario, Canada; i Department of Diagnostic and Interventional Radiology, Hospital for Sick Children, Toronto, Canada; j Department of Medical Imaging, Temerty Faculty of Medicine, University of Toronto, Affiliate to Ontario Fetal Centre, Canada; k Fetal Medicine Unit, Liverpool Women’s Hospital, Liverpool, UK; l Fetal Medicine Unit, St George’s Hospital, London, UK; m Department of Clinical Science, Intervention and Technology - CLINTEC, Karolinska Institutet, Stockholm, Sweden; n Centre for Fetal Medicine, Pregnancy Care and Delivery, Karolinska University Hospital, Stockholm, Sweden; o Department of Neonatology, Astrid Lindgren Childrens Hospital, Karolinska University Hospital, Stockholm, Sweden; p Fetal Medicine Unit, Department of Obstetrics and Gynaecology, Sheba Medical Centre, Gray Faculty of Medical & Health Sciences, Tel-Aviv University, Israel; q BCNatal Fetal Medicine Research Centre (Hospital Clínic and Hospital Sant Joan de Déu), Universitat de Barcelona, Barcelona, Spain; r Institut d’Investigacions Biomèdiques August Pi i Sunyer (IDIBAPS), Barcelona, Spain; s Department of Surgery and Medical-Surgical Specialities, School of Medicine and Health Sciences, Universitat de Barcelona, Barcelona, Spain; t Department of Neonatology, Hospital Clínic Barcelona, BCNatal (Barcelona Centre for Maternal, Fetal and Neonatal Medicine), Barcelona, Spain; u Maternal-Fetal Medicine Unit, Obstetrics Service, Hospital Italiano de Buenos Aires and Universidad Hospital Italiano, Buenos Aires, Argentina; v Department of Obstetrics and Gynaecology, TUM School of Medicine and Health, TUM University Hospital Rechts der Isar, Technical University of Munich, Munich, Germany; w Research Area of Fetal, Neonatal and Cardiological Science, Ospedale Pediatrico Bambino Gesù, Rome, Italy; x Division of Fetal Therapy, Department of Obstetrics and Gynaecology, Leiden University Medical Centre, Leiden, the Netherlands

**Keywords:** Acute exsanguination, Brain injury, Fetal anaemia, Monochorionic twins, Perimortem transfusion, Single fetal demise

## Abstract

**Background:**

Single fetal demise (sFD) in monochorionic (MC) twins may lead to acute exsanguination through placental anastomoses and result in brain injury in initially surviving cotwins. We aimed to evaluate the prevalence, type, severity, and factors associated with brain injury in initially surviving cotwins after sFD.

**Methods:**

In this international multicentre retrospective cohort study, all MC twin pregnancies complicated by sFD between 1 January 2000 and 1 January 2025 at 13 fetal therapy centres in Argentina, Belgium, Canada, Germany, Israel, Italy, the Netherlands, Spain, Sweden, and the United Kingdom were included. Pregnancies with sFD following laser surgery, selective feticide or twin anaemia-polycythaemia sequence were excluded. The primary outcome was severe brain injury in the initially surviving cotwin, assessed using all available fetal and/or neonatal neuroimaging. Severe brain injury was defined as cystic periventricular leukomalacia, multicystic encephalomalacia, migration disorders, severe ventriculomegaly or volume loss, acute hypoxic-ischaemic injury, arterial ischaemic infarction, high-grade intraventricular haemorrhage, non-punctate intraparenchymal or cerebellar haemorrhage.

**Findings:**

A total of 395 MC pregnancies with sFD and available neuroimaging were included. Median gestational age at diagnosis of sFD was 22.7 (interquartile range [IQR] 19.3–27.1) weeks. Severe brain injury was detected in 18% (72/395) of initially surviving cotwins. The most common type was multicystic encephalomalacia in 32% (23/72). The presumed timing of severe brain injury was antenatal in 82% (59/72), perinatal in 5% (4/72), postnatal in 10% (7/72) and indeterminate in 3% (2/72) of cases. In multivariable analysis, later gestational age at sFD (adjusted odds ratio [aOR] 1.09 per week, 95% confidence interval [CI] 1.03–1.14; p = 0.001) and fetal anaemia (aOR 2.29, 95% CI 1.24–4.25; p = 0.009) were associated with severe brain injury. In liveborn infants, earlier gestational age at birth was associated with severe brain injury (p = 0.009).

**Interpretation:**

Severe brain injury was diagnosed in one fifth of initially surviving cotwins in MC twin pregnancies after sFD and occurred predominantly antenatally, with imaging patterns consistent with hypoxic-ischaemic injury following acute exsanguination. Our findings support routine serial antenatal and postnatal neuroimaging after sFD. Earlier birth was associated with brain injury, but causality cannot be inferred because of confounding by indication. sFD alone should not prompt early delivery in clinically stable pregnancies. Prospective studies are needed to determine the optimal timing and modality of neuroimaging after sFD and evaluate management strategies including the use of intrauterine transfusion and optimal timing of delivery.

**Funding:**

European Society for Paediatric Research (ESPR), Leids Universitair Fonds (LUF), and Gratama Stichting, Strong Babies, Honors College Leiden University Medical Center, Janivo Stichting, Gisela Thier Fonds, Raynor Cerebellar Initiatives and Vaillant Stichting.

**Research in context**

**Evidence before this study**

We searched PubMed, Embase, Scopus, and Web of Science for studies published from January 1, 1999, through July 1, 2026, using the terms “monochorionic twins” and “fetal demise” with relevant synonyms, and screened reference lists of eligible studies. We included observational studies reporting neuroimaging outcomes in initially surviving monochorionic cotwins after spontaneous single fetal demise. In our recent systematic review and meta-analysis of 13 studies including 311 initially surviving cotwins, the pooled prevalence of brain injury after spontaneous single fetal demise (sFD) was 27% (95% confidence interval, 18–37%). However, available evidence was limited by small, heterogeneous studies with non-standardised neuroimaging protocols and outcome definitions.

**Added value of this study**

This is the largest international multicentre cohort study evaluating prevalence, type, severity, and factors associated with severe brain injury after single fetal demise in monochorionic twin pregnancies. In 395 pregnancies from thirteen fetal therapy centres, severe brain injury was detected in 18% (72/395) of the initially surviving cotwins, was predominantly hypoxic-ischaemic injury, and was of presumed antenatal origin in 82% (59/72) of cases. Later gestational age at fetal demise and signs of fetal anaemia were independently associated with brain injury. Earlier gestational age at birth was associated with severe brain injury restricted to liveborn infants.

**Implications of all the available evidence**

Severe brain injury occurred in one fifth of the initially surviving cotwins, with imaging patterns predominantly consistent with hypoxic-ischaemic injury following acute intertwin exsanguination through placental vascular anastomoses. Our findings support routine antenatal and postnatal neuroimaging after sFD, while the optimal timing and modality of postnatal imaging require prospective evaluation. We also recommend caution with early induction of birth after single fetal demise in clinically stable pregnancies to avoid additional prematurity related brain injury in initially surviving cotwins, while acknowledging that the association with brain injury may reflect reverse causation and confounding by indication rather than a causal effect of earlier delivery. Prospective studies are needed to establish the optimal timing and modality of neuroimaging after sFD and to evaluate management strategies, including the use of intrauterine transfusion and optimal timing of delivery.

## Introduction

Monochorionic (MC) twin pregnancies are at increased risk of complications due to the shared placental circulation, including twin–twin transfusion syndrome (TTTS), twin anaemia-polycythaemia sequence (TAPS), and selective fetal growth restriction (sFGR).^1^ Consequently, MC twin pregnancies are considered high-risk pregnancies and are associated with substantial perinatal morbidity and mortality, including single or double fetal demise (sFD, dFD).^1^ In unselected first-trimester MC cohorts, sFD occurs in approximately 6–8% of pregnancies and dFD in 8–12%.^2^, ^3^

In case of sFD, acute exsanguination through placental vascular anastomoses may occur and lead to acute hypovolaemic shock, fetal anaemia, and brain injury in initially surviving cotwins, or ultimately cotwin demise.^4^, ^5^, ^6^ The prevalence of severe brain injury in initially surviving cotwins is reported to range from 20 to 27%.^4^, ^5^, ^6^ However, most available evidence is derived from small observational cohorts, often from a single centre, with heterogeneous populations, inconsistent definitions of brain injury, and non-standardised neuroimaging protocols. As a result, little is known about the exact mechanisms and potential risk factors for brain injury.^4^, ^5^

In this international, multicentre study we aimed to evaluate the prevalence, type, and severity of brain injury, as well as potential factors associated with severe brain injury after sFD in a large cohort of MC twin pregnancies.

## Methods

### Study design, setting, and participants

In this retrospective observational study, all consecutive MC twin pregnancies with an initially surviving cotwin after sFD at ≥12 weeks of gestation were eligible. Cases were identified at 13 international fetal therapy centres in Argentina, Belgium, Canada, Germany, Israel, Italy, the Netherlands, Spain, Sweden, and the United Kingdom during centre-specific recruitment periods between 1 January 2000 and 1 January 2025 (Supplementary Table S1). Chorionicity and amnionicity were determined by first-trimester ultrasound according to established international guidelines.^7^ The prespecified study protocol (version 3.0, 14 June 2024) is provided in the Supplementary Materials.

We excluded pregnancies with sFD following selective feticide, TAPS, fetoscopic laser surgery of placental vascular anastomoses, twin reversed arterial perfusion sequence, higher-order multiple pregnancies, and cases with major congenital anomalies or without antenatal or postnatal neuroimaging. TAPS was excluded because its distinct placental angioarchitecture, characterised by few minuscule unidirectional anastomoses, makes acute exsanguination after sFD unlikely.^8^ This population was recently evaluated in an international TAPS Registry study of 39 pregnancies with spontaneous single fetal demise, including nine with spontaneous TAPS.^8^ None of the 37 surviving cotwins had severe brain injury, supporting our rationale for excluding TAPS.^8^ Pregnancies with fetal demise after fetoscopic laser surgery were excluded, as acute exsanguination is less likely to occur after laser, unless large anastomoses are missed. MC twin pregnancies treated with selective feticide were similarly excluded.^4^ These exclusions aimed to obtain a homogeneous cohort with a comparable underlying pathophysiological mechanism of brain injury. This cohort includes 88 cases previously published by Lanna et al. (n = 43) and van Klink et al. (n = 45).^9^, ^10^ These previous studies reported outcomes from individual centres, whereas the present study re-evaluated these cases using harmonised definitions and neuroimaging classifications as part of a substantially larger international cohort.

### Ethics

The study was reviewed by the Non-WMO Committee, Division 3, Leiden University Medical Center, and was deemed not subject to the Dutch Medical Research Involving Human Subjects Act (reference 23–3065). Ethics review and informed-consent requirements for each participating centre are provided in Supplementary Table S2. Written informed consent was obtained where required, waivers or determinations that consent was not required were applied in accordance with local requirements. The study was conducted in accordance with the Declaration of Helsinki and the General Data Protection Regulation and adhered to the Strengthening the Reporting of Observational Studies in Epidemiology reporting guideline (STROBE).

### Obstetric characteristics

The following maternal and obstetric characteristics were collected: fetal sex, gestational age (GA) at diagnosis of sFD; presumed cause of fetal demise; presence of TTTS, sFGR, and monoamniotic pregnancy, including GA at diagnosis of the MC-related complication; termination of pregnancy of the surviving twin (and indication); treatment with intrauterine transfusion (IUT); mode of birth, and reason for preterm birth (fetal, maternal, or spontaneous); GA at birth; interval between sFD and birth (days) and prenatal corticosteroid administration. Signs of fetal anaemia were defined as middle cerebral artery peak systolic velocity (MCA-PSV) ≥1.5 multiples of the median (MoM) within two weeks after sFD (measurement obtained closest to time of sFD diagnosis). Cases in which the exact MCA-PSV value was unavailable were included when the medical record documented an MCA-PSV ≥ 1.5 MoM as the indication for IUT. TTTS prior to sFD was defined according to standard ultrasound criteria.^11^ sFGR was defined as intertwin estimated fetal weight or birthweight discordance of ≥20% and classified according to Gratacós.^12^, ^13^ Presumed cause of sFD was assigned based on established diagnostic criteria and the clinical course.^7^ When MC-related complications coexisted, one primary cause was selected as the condition considered most directly related to the fetal demise. Preterm birth was categorised as moderate preterm (32 to <37 weeks), very preterm (28 to <32 weeks) or extremely preterm (<28 weeks). Centre-specific obstetric management after single fetal demise was collected from all participating centres (Supplementary Table S3). Data on race and ethnicity were not available in our dataset and could therefore not be included.

### Neuroimaging and brain injury

Local investigators classified neuroimaging according to standardised definitions of severe brain injury. Uncertain findings were additionally reviewed with the initiating centre, blinded to clinical outcome, and resolved by consensus. Severe brain injury was categorised as either diffuse or focal, based on the classification proposed by Conte and Spruijt et al.^14^, ^15^ Diffuse severe brain injury was divided into cystic periventricular leukomalacia (cPVL), multicystic encephalomalacia, migration disorders, ventriculomegaly (VM) and volume loss, or acute hypoxic-ischaemic brain injury (near time of birth, defined as perinatal origin).^14^ VM was defined as an atrial diameter ≥15 mm on fetal ultrasound (US), fetal and/or postnatal magnetic resonance imaging (MRI) or a ventricular index above the 97th percentile on neonatal cranial ultrasound.^14^ Severe focal brain injury was defined as arterial ischaemic infarction (acute or chronic stage), high-grade intraventricular haemorrhage (IVH) defined as grade III or any grade of IVH complicated by periventricular haemorrhagic infarction or post-haemorrhagic ventricular dilatation (PHVD), or non-punctate cerebellar or cerebral parenchymal haemorrhage. VM was termed PHVD if preceded by IVH ≥ grade 2. The prespecified classification was refined during data harmonisation to include acute hypoxic-ischaemic injury around birth as a separate category. The presumed timing of brain injury was classified as antenatal, perinatal, postnatal or indeterminate based on imaging timing, injury pattern and evolution.^14^, ^15^, ^16^ Antenatal injury was defined as injury detected on fetal neuroimaging after sFD, or as established cystic evolution, tissue loss or encephalomalacia on early postnatal imaging, consistent with a timing before birth. Perinatal injury was defined as injury around delivery related to perinatal asphyxia, compatible with acute hypoxic-ischaemic injury.^16^ Postnatal injury was defined as injury first detected after birth, after previously normal antenatal and/or early postnatal imaging, and supported by imaging findings typical of preterm brain injury. Indeterminate was defined when available imaging, including timing, pattern and evolution, was insufficient for reliable classification of origin. Centre-specific neuroimaging protocols after single fetal demise were collected for all participating centres (Supplementary Table S4).

### Neonatal characteristics

Postnatal characteristics included: birthweight, small-for-gestational age (SGA, birthweight < 10th percentile, according to locally used growth charts, which varied across centres), 5-min Apgar score, neonatal morbidity, and neonatal death. Severe neonatal morbidity was defined as at least one of the following: perinatal asphyxia (5-min Apgar score ≤5, need for resuscitation, mechanical ventilation for ≥10 min after birth, or cord or early neonatal blood pH < 7.0, base excess <−16 mmol/L, or lactate >10.0 mmol/L within 1 h after birth); respiratory distress syndrome requiring surfactant therapy or mechanical ventilation; neonatal sepsis (positive blood culture); necrotizing enterocolitis (Bell stage ≥ II); retinopathy of prematurity (stage ≥3); or bronchopulmonary dysplasia, defined as supplemental oxygen for at least 28 days with continued oxygen and/or respiratory support at 36 weeks’ postmenstrual age.^17^

### Statistical analysis

Continuous variables were assessed for normality by visual inspection of histograms and Q–Q plots and presented as mean with a 95% confidence interval (CI) or median with interquartile range (IQR). Categorical variables were reported as frequencies and percentages. Group comparisons were made using independent t-tests or Mann–Whitney U tests for continuous data, and Chi-square or Fisher’s exact tests for categorical data, depending on expected cell counts. No formal a priori sample-size calculation was performed because the sample size was determined by all eligible pregnancies available across participating centres during the study period.

The primary outcome for this report was severe brain injury, and the evaluation of potentially associated factors was prespecified in the study protocol. Covariates were selected a priori based on clinical relevance and previous literature and included GA at sFD, TTTS, sFGR, monoamnionicity, fetal anaemia, and fetal sex.^4^, ^9^, ^10^, ^18^ Modelling strategy and all sensitivity and exploratory analyses were not specified in the protocol and were therefore considered post-hoc. Missing covariates were handled using multiple imputation by chained equations (50 imputations; 20 iterations). The outcome, all covariates, and participating centre were included in the imputation models, and estimates were pooled using Rubin’s rules. Associations were estimated using mixed-effects logistic regression with centre as a random intercept and reported as adjusted odds ratios (aORs) with 95% CIs. Sensitivity analyses repeated the primary model in complete cases and, separately, restricted the outcome to severe brain injury of antenatal origin, using only predictor variables documented before injury detection. To assess whether the results were sensitive to a possible missing-not-at-random mechanism for fetal anaemia, delta-adjusted analyses multiplied the conditional odds of fetal anaemia for originally missing values by 0.5 and 2.0 relative to the primary imputation model. Among liveborn infants, GA at birth was examined separately using adjusted restricted cubic splines with multiple imputation and knots at the 10th, 50th, and 90th percentiles. As a post-hoc sensitivity analysis, we estimated the prevalence of severe brain injury among liveborn infants under hypothetical assumptions that 0–15% of infants without documented postnatal neuroimaging had undetected severe brain injury. Linearity was assessed using quadratic terms, multicollinearity using variance inflation factors, and potential separation and model stability using sparse-cell counts, coefficient estimates, standard errors, fitted probabilities, and convergence. Composite outcomes were classified hierarchically as neonatal death, severe brain injury, severe neonatal morbidity, or no severe adverse outcome. Analyses used SPSS 28.0 (IBM Corp., Armonk, NY, USA) and MATLAB R2022b (Version 2022b, MathWorks Inc); tests were two-sided with p < 0.05.

### Role of the funding source

Funders of the study had no role in study design, data collection, data analysis, data interpretation, or writing of the report.

## Results

A total of 445 eligible MC twin pregnancies with an initially surviving cotwin after sFD were collected from thirteen different institutions. After excluding 11% (50/445) of the cases without antenatal or postnatal neuroimaging, 395 pregnancies were included in the final analysis (Fig. 1, Supplementary Table S5). The study population included 82% (323/395) liveborn infants after sFD, 10% (38/395) cases of demise of the initially surviving cotwin, and 8% (34/395) terminations of pregnancy. In 76% (26/34) pregnancy was terminated due to imaging findings suggesting fetal brain injury. Median GA at live-birth was 36.1 weeks (IQR: 32.5–38.0, Supplementary Table S6). GA at birth was available for 88% (285/323) of liveborn infants, 8% (24/285) were born extremely preterm, 13% (37/285) very preterm, 37% (106/285) moderate preterm, and 41% (118/285) around term (≥37 weeks, Supplementary Fig. S1). Prenatal glucocorticoids were administered to 73% (33/45) of infants born before 32 weeks with available data, including 65% (11/17) of extremely preterm births and 79% (22/28) of very preterm births. Five percent (16/323) of infants died during neonatal period, including nine infants who died after redirection of care due to severe brain injury diagnosed postnatally.

**Fig. 1:**
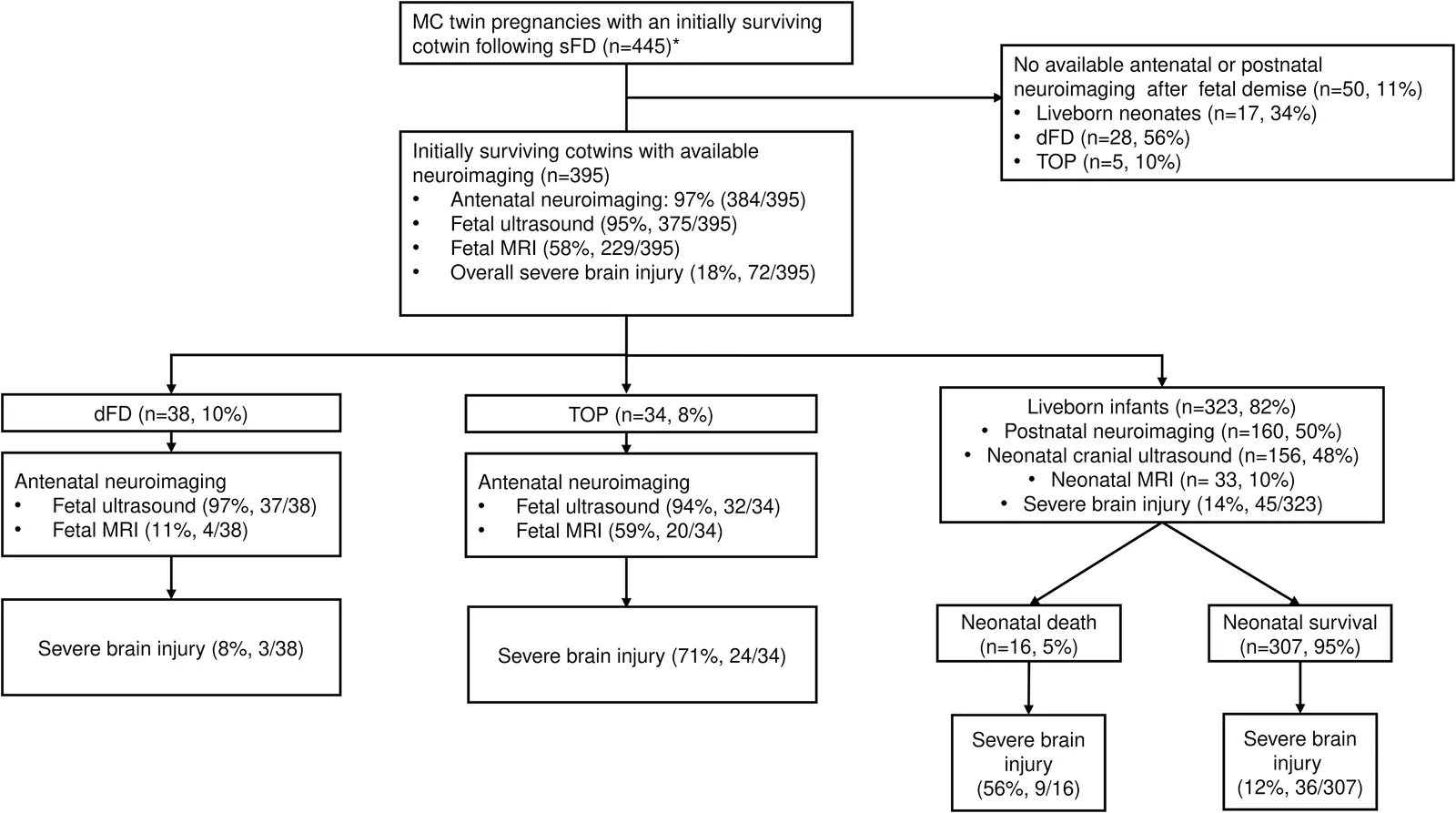
**Study profile.** ∗Prior to data collection pregnancies with single fetal demise (sFD) following selective feticide, twin anaemia-polycythaemia sequence (TAPS), fetoscopic laser surgery, twin reversed arterial perfusion sequence, higher-order multiple pregnancies, and cases with major congenital anomalies were excluded. Abbreviations: dFD, double fetal demise; TOP, termination of pregnancy; MC, monochorionic; MRI, magnetic resonance imaging.

Median GA at diagnosis of sFD was 22.7 (IQR, 19.3–27.1) weeks (Table 1, Supplementary Fig. S2). In 64% (251/395) a primary presumed cause of sFD could be identified including TTTS in 33% (129/395), sFGR in 24% (94/395), and monoamnionicity in 7% (28/395). In TTTS cases, sFD occurred in 75% (84/112) in donors among cases with known donor/recipient status. In sFGR cases, sFD occurred in the growth-restricted twin in 92% (82/89) of cases where this information was available. MCA-PSV Doppler measurements within 2 weeks after sFD were available in 64% (251/395), with a median of 1.3 MoM (IQR, 1.1–1.7). Fetal anaemia status was available in 252 cases, including one additional case in which an MCA-PSV ≥1.5 MoM was documented as the indication for IUT despite the exact value being unavailable. Fetal anaemia within 2 weeks after sFD was diagnosed in 43% (108/252) of these cases and IUT was performed in 44% (47/108). The proportion of IUT performed in initially surviving cotwins with fetal anaemia varied across participating centres, ranging between 0 and 67%.

**Table 1:** Obstetric characteristics, management and pregnancy outcomes stratified by severe brain injury.

Characteristics	Total (n = 395)	Severe brain injury (n = 72)	No severe brain injury (n = 323)	p-value
Obstetric characteristics				
Median GA at sFD (weeks, IQR)^a^	22.7 (19.3–27.1)	24.7 (21.5–28.8)	22.3 (18.9–26.7)	<0.001
TTTS (n, %)	129 (33)	17 (24)	112 (35)	0.07
Median GA at diagnosis (weeks, IQR)^b^	21.0 (18.6–23.9)	23.2 (20.1–25.6)	20.8 (18.2–23.3)	0.05
Median stage at diagnosis (I–IV, IQR)^c^	I (I–III)	III (I–III)	I (I–III)	0.09
Amnioreduction (n, %)	21 (5)	5 (7)	16 (5)	0.56
sFGR (n, %)	94 (24)	20 (28)	74 (23)	0.38
Median GA at diagnosis (weeks, IQR)^d^	18.6 (16.5–21.1)	19.4 (17.9–21.9)	17.9 (16.2–21.0)	0.29
Gratacós type at diagnosis (%)				0.05
Type I	31 (33)	3 (15)	28 (38)	
Type II	26 (28)	4 (20)	22 (30)	
Type III	29 (31)	11 (55)	18 (24)	
Unknown	8 (9)	2 (10)	6 (8)	
Monoamnionicity (n, %)	34 (9)	5 (7)	29 (9)	0.58
Post-sFD assessment and management				
MCA-PSV in survivor after sFD (MoM, IQR)^e^	1.3 (1.1–1.7)	1.5 (1.1–2.1)	1.3 (1.0–1.7)	0.002
Fetal anaemia (n/N, %)	108/252 (43)	30/53 (57)	78/199 (39)	0.02
IUT (n/N, %)	47/395 (12)	14/72 (19)	33/323 (10)	0.03
Prenatal glucocorticoids of infants born <32 weeks of gestation (n/N, %)	33/45 (73)	8/12 (67)	25/33 (76)	0.71
Caesarean delivery (n/N, %)	131/322 (41)	21/44 (48)	110/278 (40)	0.43
Pregnancy outcomes				
dFD (n, %)	38 (10)	3 (4)	35 (11)	0.08
Median GA at diagnosis (weeks, IQR)^f^	22.9 (19.0–26.5)	23.9 (23.6–26.0)	22.7 (18.6–26.9)	0.40
TOP (n, %)	34 (9)	24 (33)	10 (3)	<0.001
Median GA at TOP (weeks, IQR)	25.6 (20.8–28.9)	27.1 (24.0–29.9)	19.9 (18.7–21.9)	<0.001

Abbreviations: dFD, double fetal demise; GA, gestational age; IUT, intrauterine transfusion, IQR, interquartile range; MCA-PSV, middle cerebral artery peak systolic velocity; MoM, multiples of the median; SD, standard deviation; sFD, single fetal demise; sFGR, selective fetal growth restriction; TOP, termination of pregnancy; TTTS, twin–twin transfusion syndrome.

Data are presented as n (%) or median (IQR). Denominators vary per variable due to missing data and are indicated in the table as n/N (%) or with footnotes.

a GA at sFD was available in 393/395 (99%) pregnancies.

b GA at TTTS diagnosis was available in 124/129 (96%) TTTS pregnancies.

c Quintero stage was available in 80/129 (62%) TTTS pregnancies.

d GA at sFGR diagnosis was available in 92/94 (98%) sFGR pregnancies.

e MCA-PSV after sFD was available in 251/395 (64%) pregnancies.

f GA at dFD was available in 37/38 (97%) dFD cases.

Antenatal neuroimaging after sFD was performed in 97% (384/395) of pregnancies, including fetal ultrasound in 95% (375/395) and fetal MRI in 58% (229/395). Among the 323 liveborn neonates, postnatal neuroimaging was performed in 50% (160/323), including cranial ultrasound in 48% (156/323) and neonatal MRI in 10% (33/323). The proportion with documented postnatal neuroimaging differed across gestational-age categories and centres (centre range 16.4–100%, both p < 0.0001, Supplementary Table S7). Severe brain injury was detected in 18% (72/395) of initially surviving cotwins with available neuroimaging, and 14% (45/323) of liveborn infants, and in 12% (36/307) of neonatal survivors (Fig. 2, Supplementary Fig. S3). Sensitivity analyses showed severe brain injury in 20% (30/149) of liveborn infants with both antenatal and postnatal imaging and estimated prevalence ranged between 14 and 21% under assumptions of 0–15% undetected injury (Supplementary Table S8). The presumed origin was antenatal in 82% (59/72), perinatal in 5% (4/72), postnatal (due to prematurity) in 10% (7/72) and indeterminate in 3% (2/72) of cases.

**Fig. 2:**
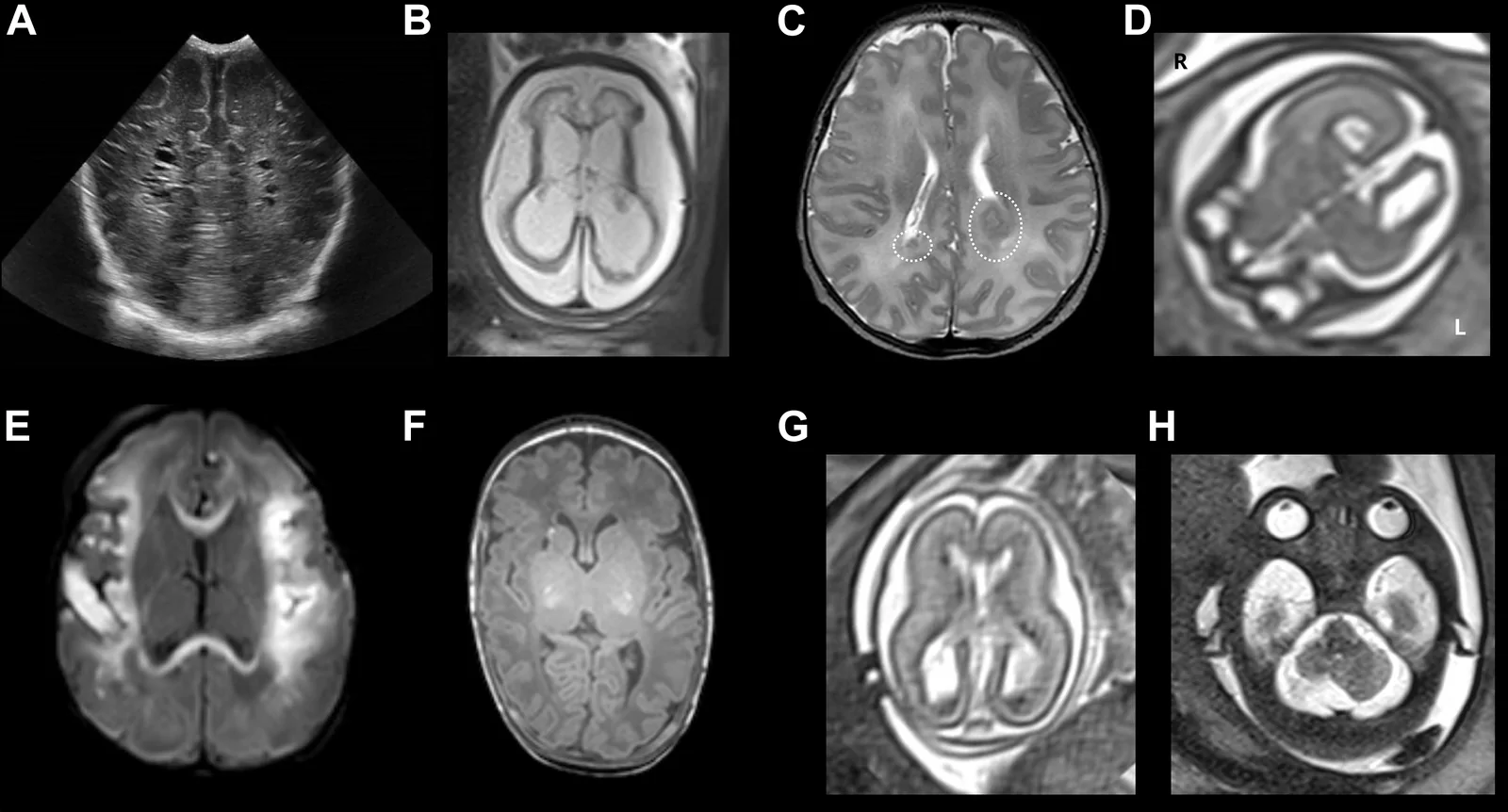
**Brain injury classification system.** Different types of brain injury in monochorionic (MC) twins after single fetal demise. Group 1 (A), cystic periventricular leukomalacia: neonatal cranial ultrasound at 28 weeks of postmenstrual age (PMA), showing extensive periventricular cysts (grade III); Group 2 (B), multicystic encephalomalacia: fetal T2-weighted magnetic resonance imaging (MRI) at 31 weeks of gestation, showing severe diffuse brain tissue loss with cortical thinning and ventriculomegaly; Group 3 (C), cortical migration disorder: neonatal T2-weighted MRI at 39 weeks PMA, showing bilateral focal heterotopia (left more pronounced); Group 4 (D), ventriculomegaly (VM) and/or volume loss: fetal T2-weighted MRI at 24 weeks of gestation, showing marked asymmetric parenchymal loss predominantly in the left hemisphere with ventriculomegaly predominantly involving the occipital horn of the left lateral ventricle. Group 5 (E), acute hypoxic ischaemic injury (around time of birth): neonatal diffusion-weighted MRI at 35 weeks PMA, showing extensive bilateral areas of restricted diffusion involving the cortex and subcortical white matter and corpus callosum. Group 6 (F), arterial ischaemic infarction: neonatal T1-weighted MRI at 32 weeks PMA, showing right-sided infarction of the caudate nucleus with secondary cystic changes and gliosis; Group 7 (G), intraventricular haemorrhage with post-haemorrhagic ventricular dilatation: fetal T2-weighted MRI at 20 weeks gestation, showing bilateral intraventricular haemorrhage with ventricular dilatation; Group 8 (H), cerebellar or cerebral parenchymal haemorrhage: fetal T2-weighted MRI at 31 weeks gestation, showing chronic stage of a right cerebellar haemorrhage, with volume loss and asymmetry of the affected right cerebellar hemisphere.

Severe brain injury was first diagnosed by fetal neurosonography in 35% (25/72) of cases, followed by fetal MRI in 35% (25/72), neonatal cranial ultrasound in 26% (19/72), and neonatal MRI in 4% (3/72, Fig. 3). Notably, 5% (3/59) of initially surviving cotwins with severe antenatal brain injury had normal fetal neurosonography findings, with the injury subsequently detected on fetal MRI. Median GA at diagnosis of severe brain injury was 27.1 weeks (IQR, 23.6–30.3).

**Fig. 3:**
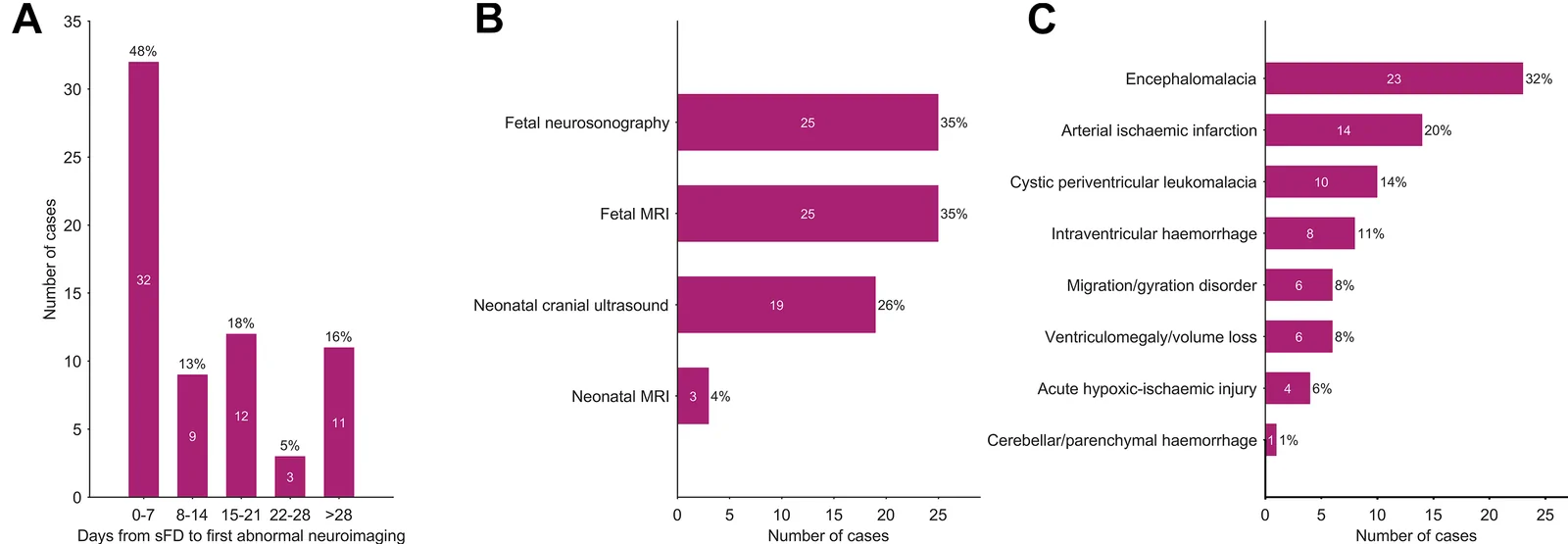
**Timing of injury, imaging modality at first detection, and spectrum of severe brain injury.** Time from single fetal demise (sFD) to first abnormal neuroimaging findings (A). Timing was available in 93% (67/72) of the cases. Imaging modality at first detection of abnormal neuroimaging findings after sFD (B). Type of brain injury of initially surviving cotwins (C). Migration disorders included lissencephaly, polymicrogyria, schizencephaly, and gray matter heterotopia (clinically significant). Abbreviations: cPVL, cystic periventricular leukomalacia; cUS, cranial ultrasound; US, ultrasound; MRI, magnetic resonance imaging.

The most common type of severe brain injury was multicystic encephalomalacia, occurring in 32% (23/72), followed by arterial ischaemic infarction in 20% (14/72), cPVL in 14% (10/72), and high-grade IVH in 11% (8/72). Migration disorders, severe VM and/or volume loss each occurred in 8% (6/72), whereas perinatal acute hypoxic-ischaemic injury around the time of birth was observed in 6% (4/72), and cerebellar or parenchymal haemorrhage in 1% (1/72).

Median GA at sFD was higher in initially surviving cotwins with severe brain injury than in those without severe brain injury, 24.7 weeks (IQR 21.5–28.8) versus 22.3 weeks (IQR 18.9–26.7, p < 0.001). MCA-PSV within 2 weeks after sFD was higher in initially surviving cotwins with severe brain injury, 1.5 MoM (IQR 1.1–2.1) versus 1.3 MoM (IQR 1.0–1.7, p = 0.002). Initially surviving cotwins with signs of fetal anaemia showed severe brain injury in 28% (30/108) of cases compared to 16% (23/144) of initially surviving cotwins without fetal anaemia (p = 0.02). Among fetuses with anaemia, severe brain injury occurred in 29.8% (14/47) with IUT and 26.2% (16/61) with expectant management (p = 0.68). Median interval between sFD and liveborn birth was shorter in initially surviving cotwins with severe brain injury, 3.4 weeks (IQR 0.1–9.5) versus 12.7 weeks (IQR 4.9–18.1, p < 0.001, Supplementary Fig. S4).

Severe brain injury occurred in 33% (14/42) of the liveborn infants born within 1 week after sFD, with an antenatal origin in 50% (7/14), perinatal origin in 29% (4/14), and postnatal origin in 21% (3/14). A maternal indication for delivery was present in 10% (4/42) of cases, while a fetal indication was documented in 69% (29/42), most commonly fetal distress (n = 15), fetal anaemia (n = 6), or concern about further acute exsanguination (n = 6). In 21% (9/42), no specific maternal or fetal indication for delivery was documented. Median interval between sFD and live birth across countries ranged from 2.6 to 17.4 weeks, with a median of 11.6 weeks (IQR 3.6–17.9, Supplementary Fig. S5).

In univariable analyses, later GA at sFD (OR 1.08 per week, 95% CI 1.03–1.13; p = 0.002) and fetal anaemia (OR 2.02, 1.10–3.74; p = 0.025) were associated with severe brain injury. In the primary multiple-imputation mixed-effects model (n = 395; 72 events), later GA at sFD (aOR 1.09, 1.03–1.14; p = 0.001) and fetal anaemia (aOR 2.29, 1.24–4.25; p = 0.009) remained associated, whereas the other prespecified factors were not (Fig. 4, Supplementary Table S9). The association with fetal anaemia remained robust across the delta-adjusted missing-not-at-random sensitivity analyses. The quadratic term was non-significant (p = 0.10), maximum variance inflation factor was 1.11, the smallest predictor–outcome cell contained five cases, and all 50 imputed models converged without instability.

**Fig. 4:**
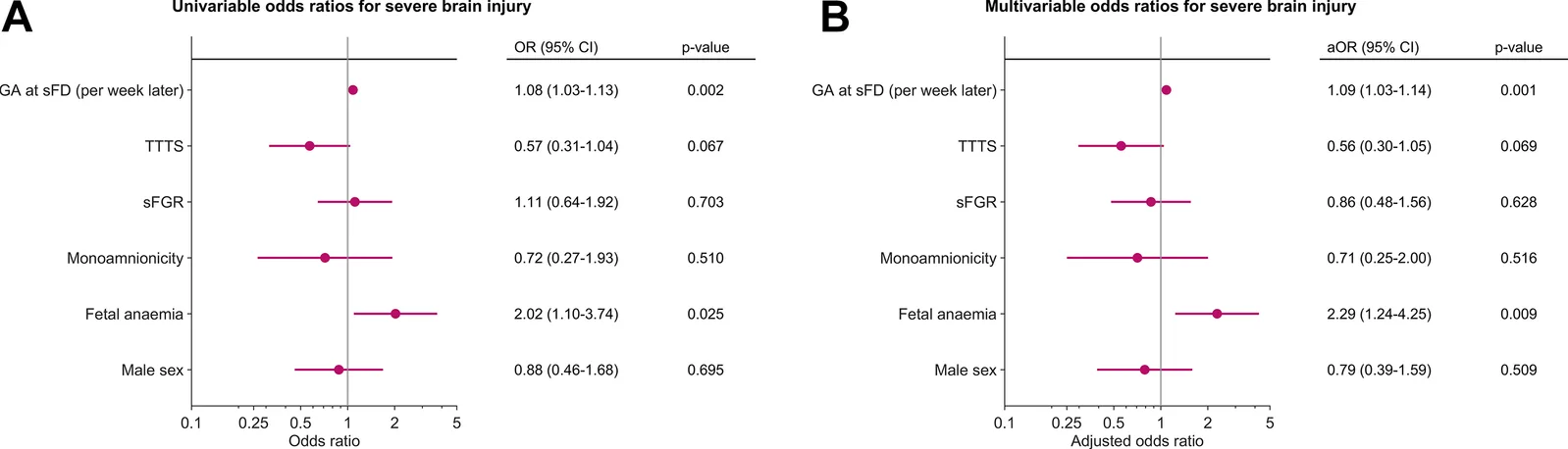
**Univariable and multivariable logistic regression of factors associated with severe brain injury after single fetal demise.** Forest plots show univariable odds ratios (ORs; A) and adjusted odds ratios (aORs; B) with 95% confidence intervals (CIs). The primary multivariable analysis used multiple-imputation mixed-effects logistic regression including gestational age (GA) at single fetal demise (sFD), twin–twin transfusion syndrome (TTTS), selective fetal growth restriction (sFGR), monoamnionicity, fetal anaemia, and fetal sex, with participating centre as a random intercept. The vertical line indicates the null value (OR/aOR = 1). For gestational age at sFD, effect estimates are expressed per one-week later occurrence of sFD.

Complete-case estimates were directionally similar but less precise (n = 188; 32 events; Supplementary Fig. S6). In the antenatal-origin sensitivity analysis (n = 382; 59 events), later GA at sFD (aOR 1.06, 1.00–1.12; p = 0.034) and fetal anaemia (aOR 2.47, 1.27–4.81; p = 0.008) were associated with severe brain injury, TTTS showed an inverse association (aOR 0.49, 0.24–0.98; p = 0.044; Supplementary Fig. S7). Among liveborn infants, exploratory spline analysis showed an overall association between GA at birth and severe brain injury (p = 0.009), without evidence of non-linearity (p = 0.52; Supplementary Fig. S8).

Neonatal death occurred in 19.0% (8/42), 14.3% (5/35), and 1.4% (3/208) of infants born <1 week, 1–<4 weeks, and ≥4 weeks after sFD, respectively. The corresponding proportions were 19.0% (8/42), 20.0% (7/35), and 7.7% (16/208) for survival with severe brain injury, and 35.7% (15/42), 34.3% (12/35), and 11.1% (23/208) for severe neonatal morbidity without neonatal death or severe brain injury (Fig. 5).

**Fig. 5:**
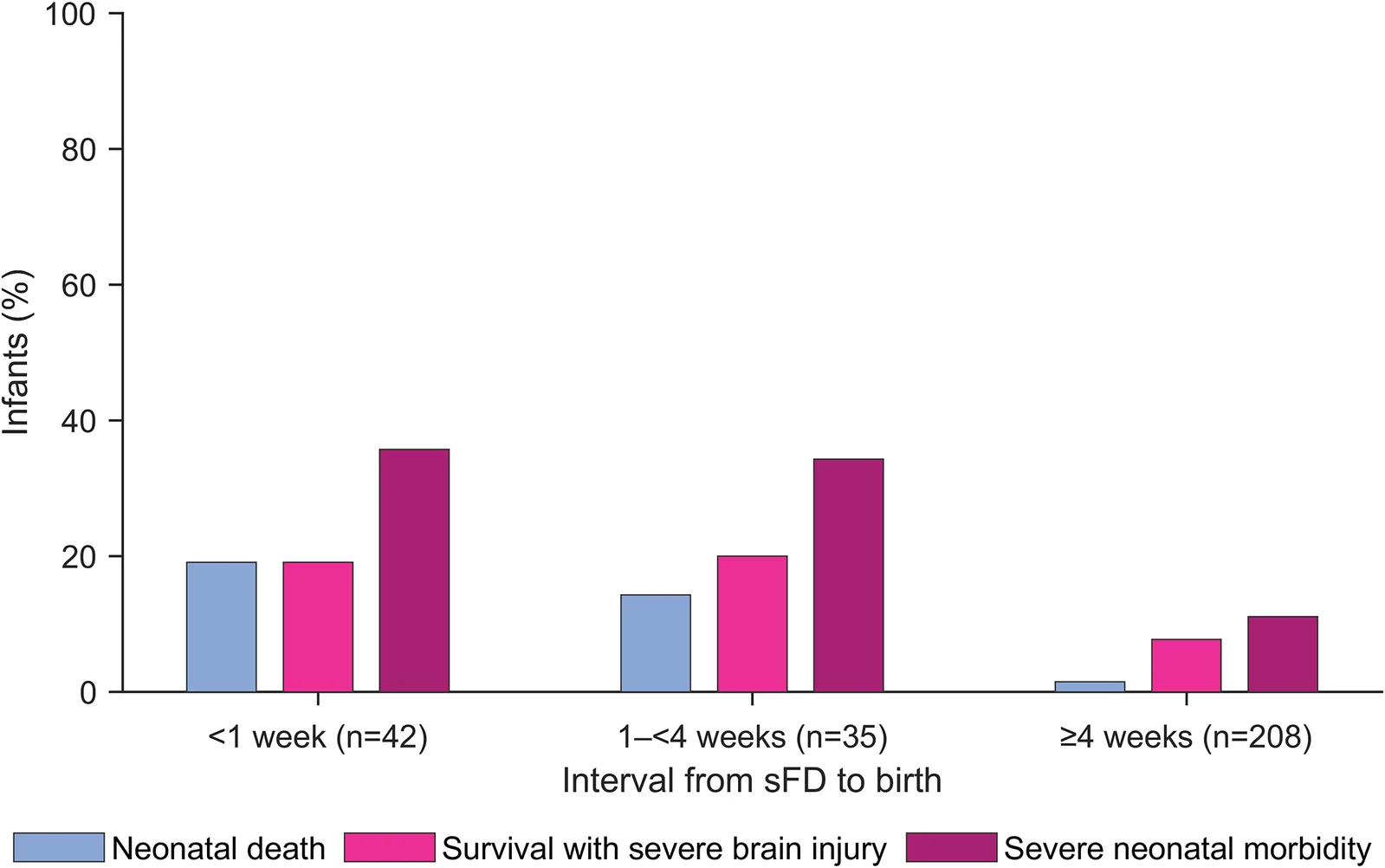
**Hierarchical outcomes by interval from single fetal demise to liveborn birth.** Percentage of infants with severe neonatal morbidity, severe brain injury, and neonatal death stratified by interval (<1 week, 1 to <4 weeks, ≥4 weeks) between single fetal demise (sFD) and birth. Each infant was assigned once according to the most severe outcome: neonatal death (NND), severe brain injury, severe neonatal morbidity without severe brain injury, or no severe adverse neonatal outcome. Bar heights represent the percentage of infants within each interval category. Numbers in parentheses indicate the number of infants per group.

## Discussion

This large multicentre cohort study showed that one fifth of initially surviving cotwins of MC pregnancies complicated by sFD exhibit severe brain injury, predominantly of antenatal origin. The observed pattern of antenatal severe brain injury, primarily multicystic encephalomalacia, cPVL, and arterial ischaemic infarction, is consistent with hypoxic-ischaemic brain injury following acute exsanguination and hypovolaemic shock.^16^ In accordance with international guidelines, routine antenatal neuroimaging, including fetal neurosonography and MRI of the initially surviving cotwins within 4–6 weeks after demise is therefore strongly recommended.^7^ Importantly, one third of abnormalities were first identified on postnatal neuroimaging, although postnatal imaging was performed in only half of liveborn infants. This finding highlights an important gap in neonatal assessment and supports routine postnatal neuroimaging after sFD. Postnatal imaging availability and clinical practice nevertheless varied between centres and over time.

A recent systematic review and meta-analysis reported a prevalence of brain injury of 27%.^4^ However, less stringent definitions of severity were used, which may explain the slightly higher rate of brain injury compared with our cohort. Rates of neonatal death were similar between studies (6% versus 5%), whereas termination of pregnancy was more frequently reported in our cohort (9% versus 3%). This difference may be explained by changes in prenatal neuroimaging over the extended inclusion period, as well as variation in country-specific legal frameworks, clinical practices, ethical approaches to termination of pregnancy, and increased awareness of clinicians.^4^ The lower proportion of postnatally acquired severe brain injury in our cohort (10% versus 32%) may partly be explained by the lower proportion of liveborn infants with severe brain injury who were born very or extremely preterm (42% versus 62%), although methodological differences between studies should also be considered.^4^ Another recent meta-analysis reported cerebral abnormalities in 20%, although direct comparison is limited by broader eligibility criteria that did not consistently account for whether acute intertwin exsanguination through bidirectional anastomoses was pathophysiologically possible.^5^

The substantial variation between centres in the interval from sFD to liveborn birth indicates marked heterogeneity in clinical management and international guidance. This variation likely reflects both clinical uncertainty and gaps in evidence-based guidance. Although this observational study cannot determine the optimal use of IUT or timing of delivery because of confounding by indication, it quantifies current practice variation and identifies priorities for prospective research and international consensus. The International Society of Ultrasound in Obstetrics and Gynecology (ISUOG) and International Federation of Gynecology and Obstetrics (FIGO) advise against immediate delivery in preterm MC twins after sFD, with ISUOG considering delivery at 34–36 weeks after a course of maternal steroids, whereas Royal College of Obstetricians and Gynaecologists (RCOG) recommends aiming to prolong gestation until term but individualising timing according to ultrasound findings, growth, maternal factors and parental preference.^7^, ^19^, ^20^ In contrast, the Society for Maternal-Fetal Medicine (SMFM) and the American College of Obstetricians and Gynecologists (ACOG) do not provide specific guidance regarding timing of delivery after spontaneous sFD.^21^

In the primary analysis, we identified later GA at sFD and fetal anaemia in the initially surviving cotwin as independent factors associated with severe brain injury, consistent with existing literature.^9^, ^10^, ^22^ The association with later GA at sFD may be explained by differences in placental angioarchitecture. As gestation advances, the diameter of superficial arterio-arterial and veno-venous anastomoses increases, resulting in lower vascular resistance (Poiseuille’s law) and predisposing to larger volume shifts in the event of acute exsanguination through the anastomoses.^23^ This mechanism is consistent with previous work showing that the presence, size and number of superficial anastomoses are significantly associated with severe brain injury after fetal demise.^24^ Alternatively, maturation-dependent differences in fetal brain vulnerability may contribute to variation in severity and pattern of hypoxic-ischaemic injury after acute haemodynamic compromise.^16^, ^25^

The association with signs of fetal anaemia likely reflects the severity of acute intertwin transfusion, with larger anastomoses facilitating larger blood loss or hypovolaemic shock and hypoxic injury related to anaemia.^8^, ^23^ In line with previous studies, an elevated MCA-PSV within 2 weeks after sFD is an important indicator of brain injury.^10^, ^22^ However, normal MCA-PSV values do not exclude brain injury.^10^ IUT may correct anaemia after sFD. One small multicentre retrospective cohort study investigated the use of IUT after sFD in a non-matched comparison and did not show benefit over expectant management.^26^, ^27^ Further in-depth evaluation of the effects of IUT in this cohort is planned separately.

Earlier GA at birth was associated with severe brain injury in an exploratory analysis restricted to liveborn infants. Importantly, neonatal morbidity was significantly more frequently observed in infants born less than 4 weeks after sFD compared with those born 4 weeks or more after sFD. Furthermore, prenatal glucocorticoids were not administered in one quarter of the very or extremely preterm infants with available data, suggesting that some preterm births occurred without adequate preparation.^28^, ^29^ These findings should be interpreted with caution because most severe brain injury was already of antenatal origin and earlier delivery was frequently prompted by fetal compromise, resulting in potential reverse causation and confounding by indication.

We hypothesise that brain injury after sFD may arise through a triple-hit mechanism. First, a proportion of brain injury may be related to the underlying complication preceding sFD, such as TTTS, sFGR or monoamnionicity, and may therefore have already been present before sFD occurred. The second hit may reflect acute haemodynamic imbalance and intertwin exsanguination resulting in hypoxic-ischaemic injury.^4^ This injury can be detected prenatally by fetal neuroimaging emphasising the importance of routine fetal ultrasound and fetal brain MRI following sFD. The residual risk of brain injury following normal fetal neurosonography and MRI is still unknown. Lastly, a third hit may occur after extreme or very preterm birth, through prematurity-related brain injury and neonatal morbidity.^4^ Although earlier delivery was associated with adverse outcome, causality cannot be inferred because of reverse causation and likely confounding by indication. In clinically stable pregnancies, expectant management with close surveillance should be aimed for to reduce prematurity-related morbidity. However, in the presence of fetal distress at viable gestations, management should be individualised, balancing the risk of extreme or very preterm birth against the risk of double fetal demise, while taking parental preferences into account.

This study has several strengths. It represents the largest cohort to date of MC pregnancies complicated by sFD. The use of standardised definitions and a predefined study protocol across centres improves the validity of the findings. In addition, inclusion of both antenatal and postnatal neuroimaging allowed assessment of the type and timing of brain injury. Finally, this international collaboration provides a foundation for future prospective studies of this rare condition. This study also has some limitations. First, the retrospective design and differential availability of neuroimaging may have introduced selection bias, while reliance on historical data resulted in missing data. Clinical management, imaging protocols, and documentation were not standardised across centres and changed over time during the study period. Although the international multicentre design enhances generalisability to tertiary fetal medicine practice, differing referral patterns and neuroimaging-based inclusion limit population-level representativeness. Second, not all initially surviving cotwins underwent antenatal and postnatal neuroimaging, as fetal MRI and/or neonatal cranial ultrasound or MRI were not routinely performed. Postnatal imaging availability also differed according to gestational age and clinical condition, introducing potential ascertainment bias. Consequently, the prevalence of postnatal brain injury may have been underestimated. Third, although neuroimaging was assessed locally using prespecified definitions across centres and uncertain findings were additionally reviewed with the initiating centre, formal independent dual review and assessment of interobserver agreement were not assessed. Fourth, missing data for fetal anaemia and fetal sex were addressed using multiple imputation, with similar effect estimates in complete-case analyses. However, this approach relies on assumptions about the missing-data mechanism that cannot be empirically verified. Fifth, pre-sFD neuroimaging was not systematically available, limiting definitive attribution of migration disorders, severe ventriculomegaly, and volume loss to the sFD event. Finally, we did not perform a separate analysis of monoamniotic pregnancies because this subgroup comprised only 9% (34/395) of the cohort and the limited number of events precluded reliable analysis. Monoamnionicity may confer additional risks related to cord entanglement after fetal demise and warrants dedicated investigation in a larger multicentre cohort with sufficient statistical power.

In conclusion, severe brain injury occurred in one fifth of initially surviving cotwins after sFD, was predominantly hypoxic-ischaemic in nature, and was independently associated with later GA at sFD and fetal anaemia at diagnosis of sFD. Most severe brain injury cases were antenatal in origin, whereas 10% were classified as postnatal and prematurity-related. Our findings support routine serial antenatal and postnatal neuroimaging after sFD. In clinically stable pregnancies, sFD alone should not prompt early delivery.

## Contributors

MR contributed to conceptualization, methodology, investigation, data curation, formal analysis, visualization, project administration, writing of the original draft, and writing, review, and editing. LL, MA, FR, ML, DC, AR, SShi, EM, LSC, AK, SSor, DM, LH, KB, YY, DAK, DSR, PR, CHM, LO, JUO, and IF contributed to investigation, data curation, resources, and writing, review, and editing. LCMZ contributed to investigation, data curation, validation, and writing, review, and editing. FS and MCH contributed to conceptualization, methodology, investigation, resources, and writing, review, and editing. JMMvK, LSdV, SGG, and SJS contributed to conceptualization, methodology, supervision, and writing, review, and editing. EL contributed to conceptualization, data curation, methodology, supervision, project administration, validation, writing of the original draft, and writing, review, and editing. MR and LCMZ accessed and verified the underlying data. MR and EL were responsible for the decision to submit the manuscript. All authors critically reviewed and approved the final version of the manuscript.

## Data sharing statement

Individual participant data, including data dictionaries, that underlie the results reported in this article (text, tables, figures, and appendices), together with the study protocol, will be available beginning 3 months and ending 10 years after publication. Data sharing requests will be considered only for researchers who provide a methodologically sound proposal. Each participating centre will independently determine whether to approve data sharing in accordance with its local ethical, legal, and data governance requirements. The coordinating centre will not share data from any participating centre without that centre’s explicit approval. Proposals should be directed to the corresponding author (m.rondagh@lumc.nl).

## Declaration of interests

LL, SGG, LH, and DSR report research grants. EL and SGG report reimbursement of travel expenses for participation as faculty at scientific meetings. LH reports serving as co-principal investigator in studies sponsored by Johnson & Johnson and membership of an educational board. SJS reports royalties and unpaid committee and data safety monitoring board roles. LSdV reports royalties and reimbursement of travel expenses. All other authors declare no competing interests.

## Appendix A Supplementary data

Supplementary Material

Supplementary study group list
