# Supplementary material for: Brain injury after single fetal demise in monochorionic twin pregnancies: an international multicentre retrospective cohort study
Source: eClinicalMedicine. 2026 Oct 28;101:104290. doi: 10.1016/j.eclinm.2026.104290 (PMC13735160; doi:10.1016/j.eclinm.2026.104290)
Supplement: Supplementary file 1 [file mmc1.pdf]

Brain Injury After Single Fetal Demise in Monochorionic Twin Pregnancies: An International Multicentre Retrospective Cohort Study

Manuscript number: eclinm-D-26-03509R1

This appendix contains supplementary tables, supplementary figures, and the CITRUS study protocol.

Table of contents

Supplementary Figures 3

Supplementary Figure 1. Hierarchical adverse neonatal outcomes according to gestational age at birth 3

Supplementary Figure 2. Gestational age at single fetal demise according to severe brain injury 4

Supplementary Figure 3. Neuroimaging follow-up in initially surviving co-twins after single fetal demise in monochorionic twin pregnancies. 5

Supplementary Figure 4. Severe brain injury according to the interval from single fetal demise to birth 6

Supplementary Figure 5. Interval between single fetal demise and birth across participating countries.. 7

Supplementary Figure 6. Comparison of the primary multiple-imputation and complete-case analyses 8

Supplementary Figure 7. Sensitivity analysis restricted to severe brain injury of antenatal origin 9

Supplementary Figure 8. Association between gestational age at birth and severe brain injury among liveborn infants 10

Supplementary Tables 11

Supplementary Table 1. Centre-specific recruitment periods and referral characteristics.. 11

Supplementary Table 2. Ethics approval and informed-consent requirements by participating centre 12

Supplementary Table 3. Centre-specific obstetric management after single fetal demise in monochorionic twin pregnancies 15

Supplementary Table 4. Centre-specific neuroimaging protocols after single fetal demise in monochorionic twin pregnancies 18

Supplementary Table 5. Obstetric characteristics, management and pregnancy outcomes of included and excluded pregnancies 21

Supplementary Table 6. Neonatal characteristics and short-term outcome of liveborn infants after single fetal demise 23
Supplementary Table 7. Documented postnatal neuroimaging by gestational age at birth and participating centre 25
Supplementary Table 8. Sensitivity analyses for incomplete postnatal neuroimaging 26
Supplementary Table 9. Sensitivity analyses of the multivariable model for severe brain injury following single fetal demise 27
Panel A. Primary, complete-case, and antenatal-origin analyses 27
Panel B. Missing-not-at-random sensitivity analysis for fetal anaemia 28
Study Protocol 29
CITRUS Study protocol 29

Supplementary Figures

Supplementary Figure 1. Hierarchical adverse neonatal outcomes according to gestational age at birth

Percentages of liveborn infants with neonatal death, survival with severe brain injury, or severe neonatal morbidity, stratified by gestational age at birth (<28 weeks, 28–<32 weeks, 32–<37 weeks, and ≥37 weeks). Outcomes were classified hierarchically as neonatal death, survival with severe brain injury, severe neonatal morbidity without severe brain injury, or no severe adverse neonatal outcome, with each infant assigned once according to the most severe outcome experienced. Gestational age at birth was available for 88% (285/323) of liveborn infants.

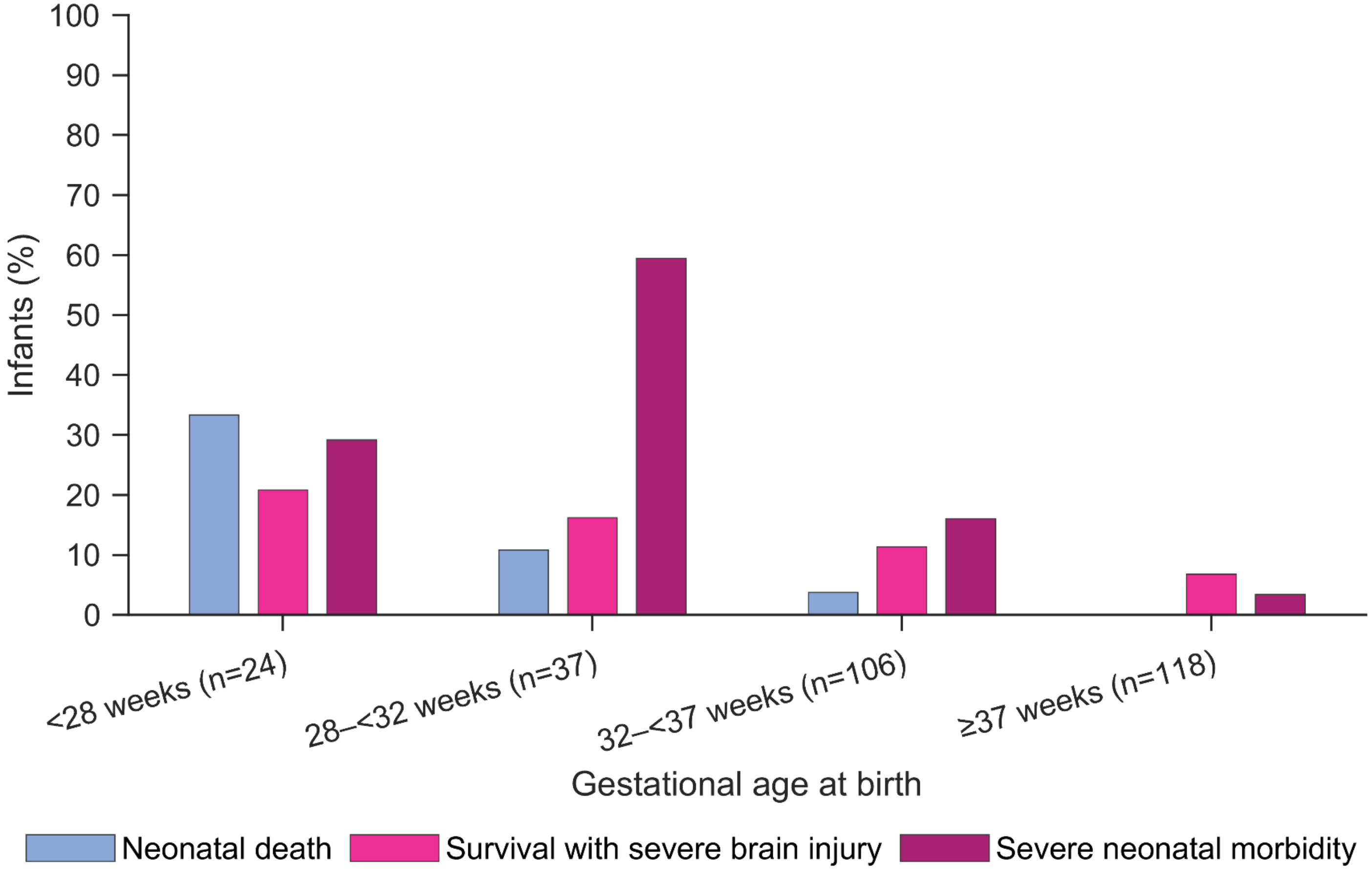

Supplementary Figure 2. Gestational age at single fetal demise according to severe brain injury

Distribution of gestational age at single fetal demise among the 395 initially surviving co-twins with an assessable severe brain injury outcome, stratified by the presence or absence of severe brain injury. Stacked bars represent the number of pregnancies within each one-week gestational-age interval. Severe brain injury occurred in 18% (72/395) of initially surviving co-twins.

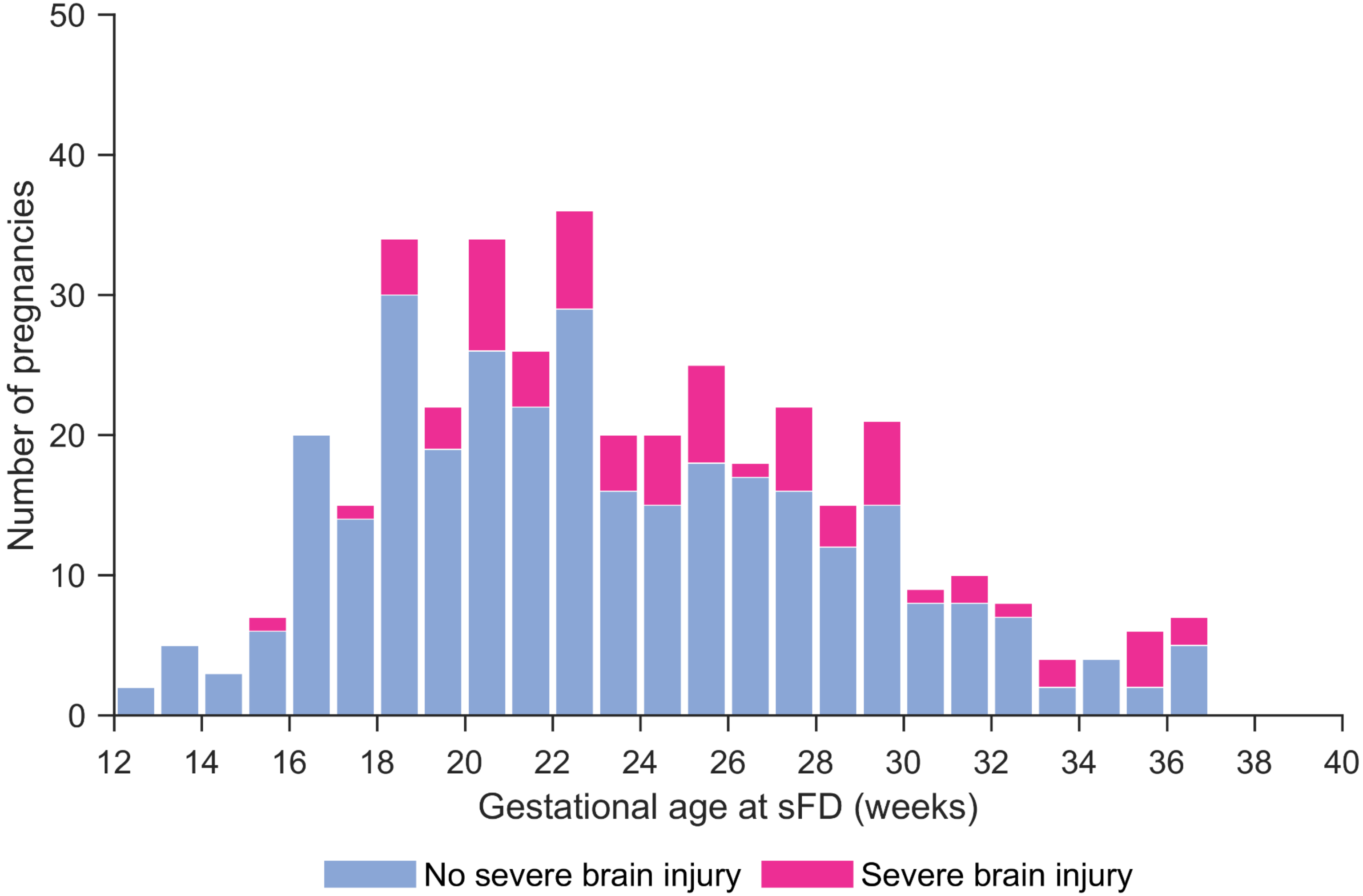

Supplementary Figure 3. Neuroimaging follow-up in initially surviving co-twins after single fetal demise in monochorionic twin pregnancies.

The upper panel shows the number of initially surviving co-twins for each observed combination of fetal neurosonography, fetal magnetic resonance imaging (MRI), neonatal cranial ultrasound, and neonatal MRI. The lower panel indicates which examinations were performed within each combination. Filled circles indicate examinations that were performed, open circles examinations that were not performed, and vertical lines connect examinations belonging to the same combination. Fetal ultrasound was performed in 95% (375/395) and fetal MRI in 58% (229/395) of initially surviving co-twins. Postnatal neuroimaging was performed in 50% (160/323) of liveborn infants, including neonatal cranial ultrasound in 48% (156/323) and neonatal MRI in 10% (33/323).

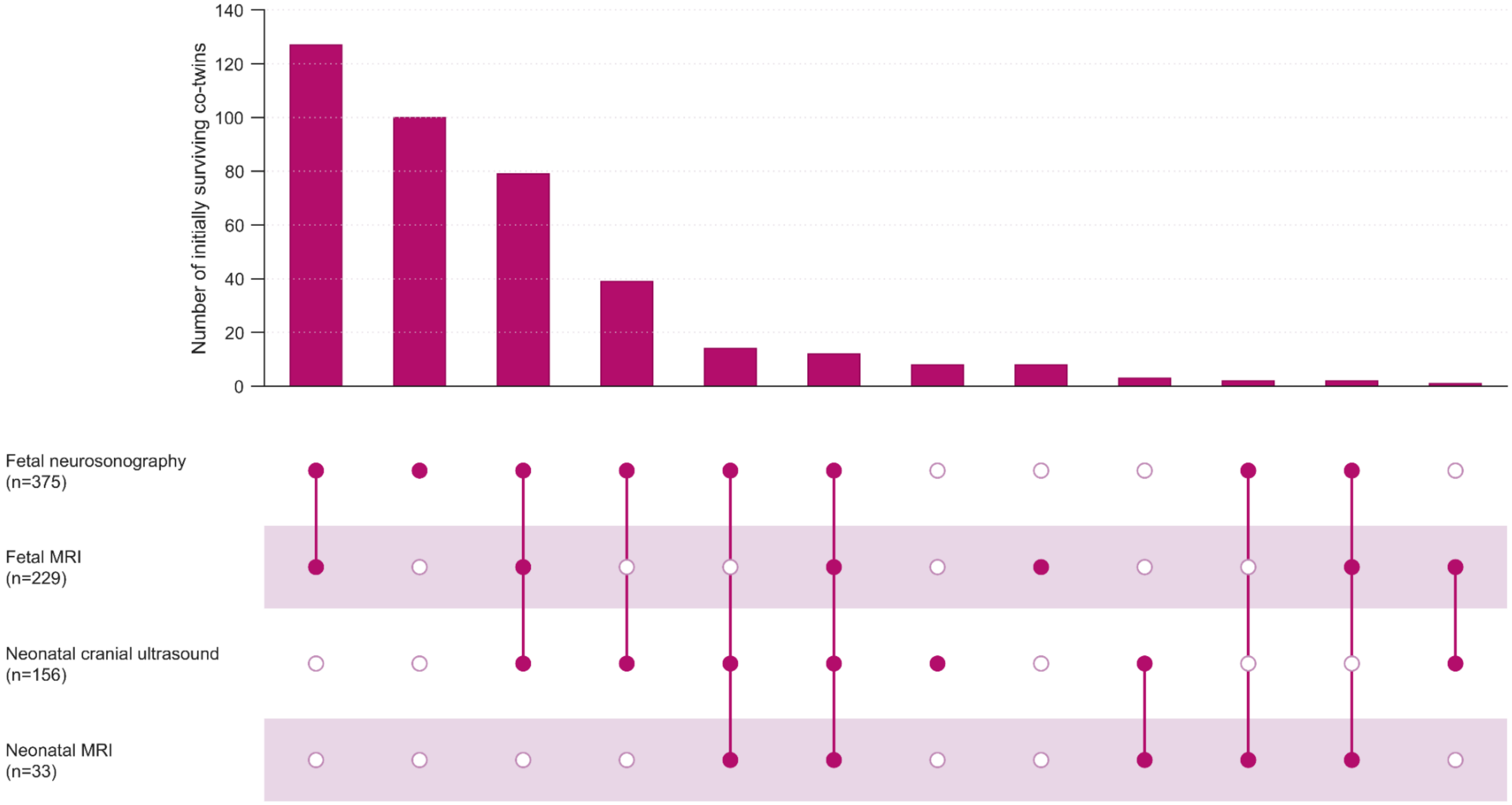

Supplementary Figure 4. Severe brain injury according to the interval from single fetal demise to birth

Bars show the percentage of liveborn infants with severe brain injury according to the interval from single fetal demise to birth: less than 1 week, 1–<4 weeks, 4–<8 weeks, and 8 weeks or longer. Severe brain injury occurred in 33% (14/42), 26% (9/35), 13% (5/38), and 7% (12/170) of infants in these respective interval categories. Interval data were available for 88% (285/323) of liveborn infants. This descriptive analysis does not establish a causal effect of delivery timing because the association may be affected by confounding by indication.

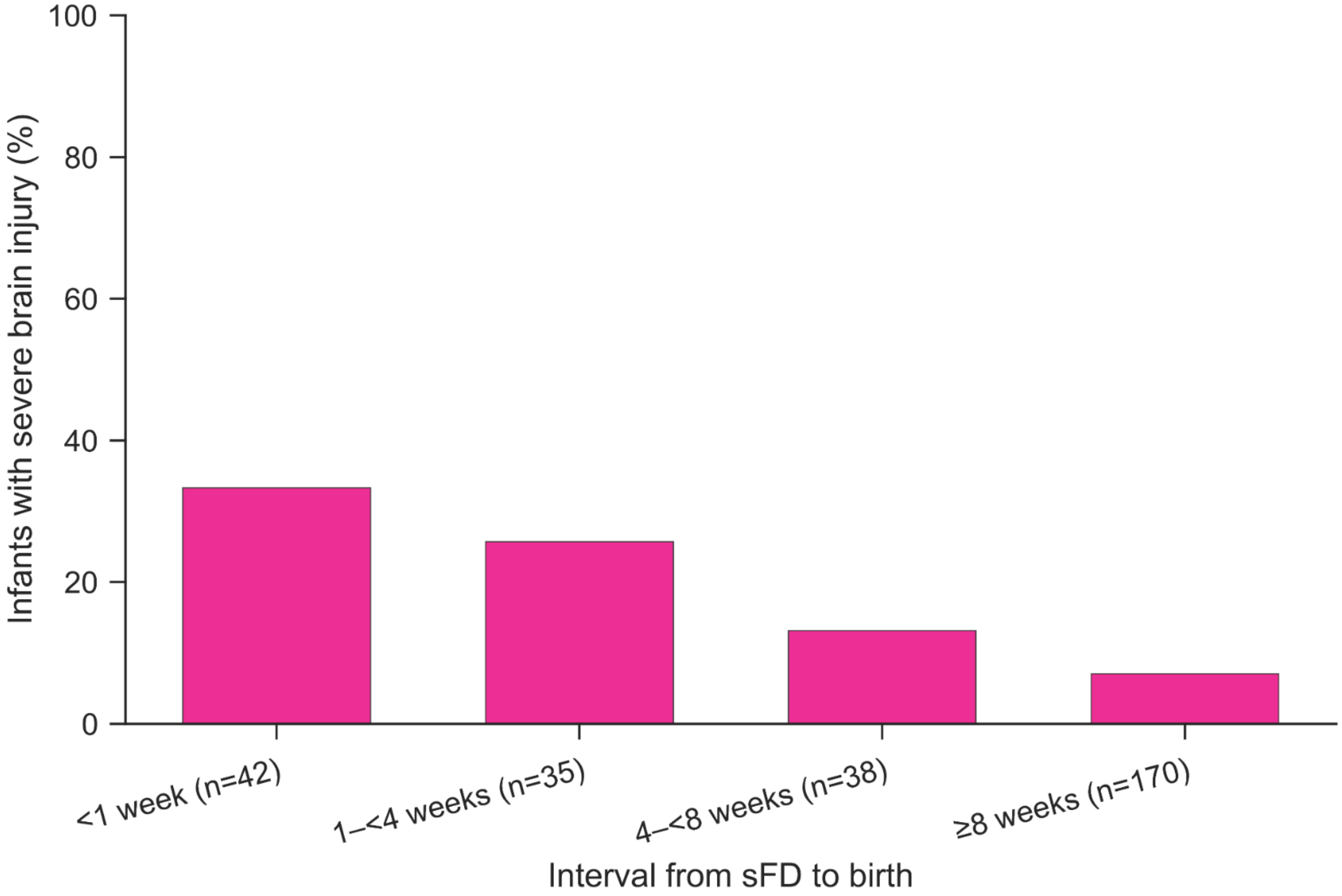

Supplementary Figure 5. Interval between single fetal demise and birth across participating countries

Pie charts show the distribution of the interval from single fetal demise to birth among liveborn infants, overall and by country, using the categories less than 1 week, 1–<2 weeks, 2–<3 weeks, 3–<4 weeks, and 4 weeks or longer. Countries are ordered by the proportion of infants born within 4 weeks after single fetal demise. The median interval and interquartile range (IQR) are displayed below each chart. Interval data were available for 88% (285/323) of liveborn infants. Availability was 44% (27/61) in Canada, 98% (40/41) in Belgium, 91% (21/23) in the United Kingdom, and 88% (7/8) in Spain. Interval data were complete for all other countries.

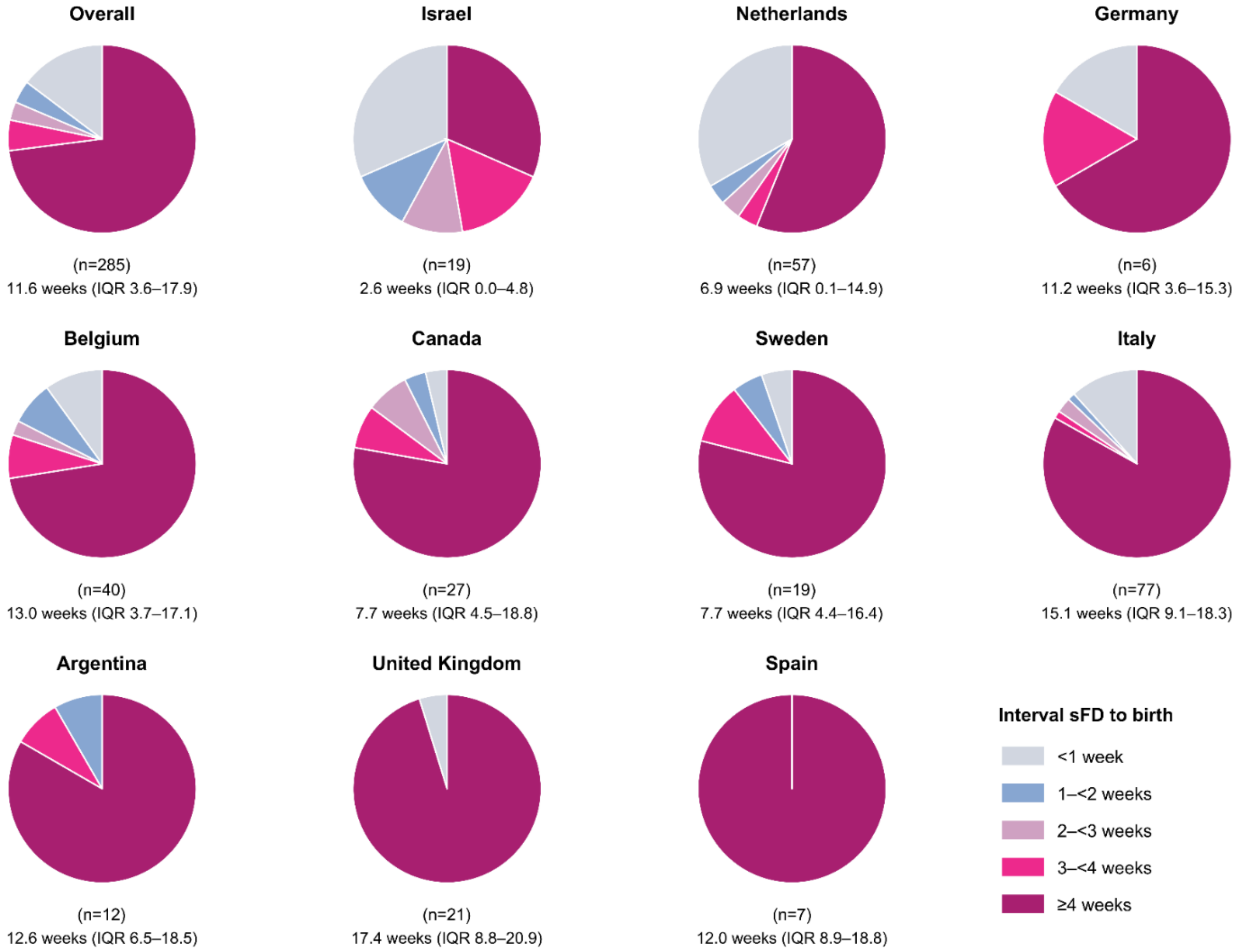

Supplementary Figure 6. Comparison of the primary multiple-imputation and complete-case analyses

Adjusted odds ratios with 95% CIs for factors associated with severe brain injury in the primary multiple-imputation analysis and complete-case sensitivity analysis. Both mixed-effects logistic regression models included gestational age at single fetal demise, twin-to-twin transfusion syndrome, selective fetal growth restriction, monoamnionicity, fetal anaemia, and fetal sex, with participating centre as a random intercept. The primary analysis used multiple imputation by chained equations with 50 imputed datasets and included 395 initially surviving co-twins with 72 severe brain injury events. The complete-case analysis used the same covariates and included 188 co-twins with 32 events. Filled circles represent the primary analysis and open squares the complete-case analysis. The vertical line indicates the null value of 1. For gestational age at single fetal demise, the adjusted odds ratio is expressed per week later. Abbreviations: GA, gestational age; sFD, single fetal demise; TTTS, twin-to-twin transfusion syndrome; sFGR, selective fetal growth restriction; CI, confidence interval.

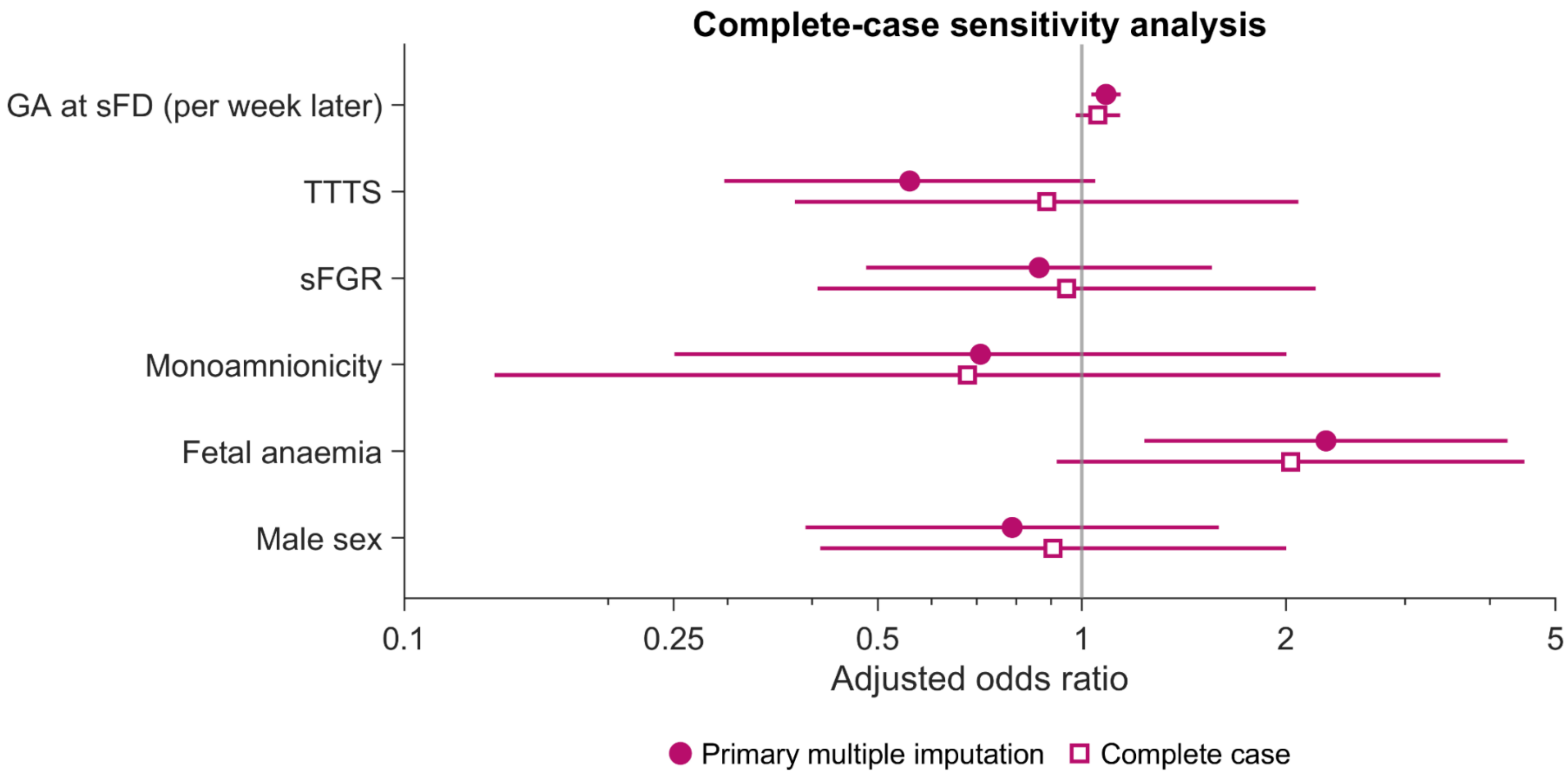

Supplementary Figure 7. Sensitivity analysis restricted to severe brain injury of antenatal origin

Adjusted odds ratios with 95% CIs from the multiple-imputation mixed-effects logistic regression model restricted to severe brain injury of antenatal origin. Antenatal origin was determined from the timing, pattern, and evolution of the neuroimaging findings according to the prespecified criteria detailed in the Methods. The model included gestational age at single fetal demise, twin-to-twin transfusion syndrome, selective fetal growth restriction, monoamnionicity, fetal anaemia, and fetal sex, with participating centre as a random intercept. The analysis included 382 initially surviving co-twins, comprising 59 with antenatal severe brain injury and 323 without severe brain injury. Four perinatal, seven postnatal, and two indeterminate severe brain injuries were excluded. The vertical line indicates the null value of 1. aOR, adjusted odds ratio; CI, confidence interval; GA, gestational age; sFD, single fetal demise; TTTS, twin-to-twin transfusion syndrome; sFGR, selective fetal growth restriction.

Sensitivity analysis restricted to antenatal severe brain injury

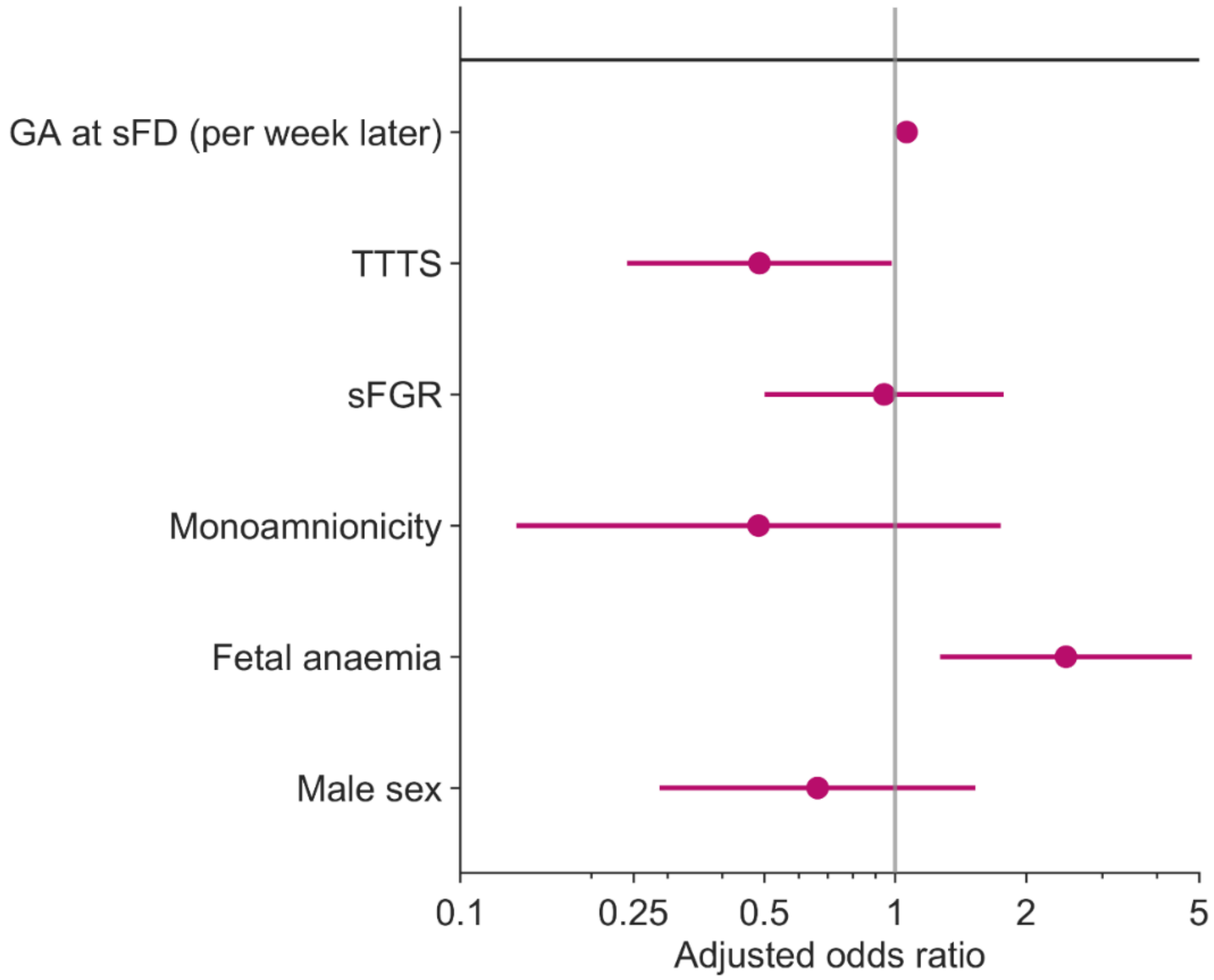

	aOR (95% CI)	p-value
GA at sFD (per week later)	1.06 (1.00-1.12)	0.034
TTTS	0.49 (0.24-0.98)	0.044
sFGR	0.94 (0.50-1.77)	0.855
Monoamnionicity	0.48 (0.13-1.75)	0.268
Fetal anaemia	2.47 (1.27-4.81)	0.008
Male sex	0.66 (0.29-1.53)	0.336

Supplementary Figure 8. Association between gestational age at birth and severe brain injury among liveborn infants

Restricted cubic spline analysis among all 323 liveborn infants, including 45 with severe brain injury, with multiple imputation of missing gestational age at birth. Knots were placed at the 10th, 50th, and 90th percentiles (28.5, 36.0, and 39.0 weeks), with the median gestational age of 36.0 weeks as the reference. The model was adjusted for gestational age at single fetal demise, twin-to-twin transfusion syndrome, selective fetal growth restriction, monoamnionicity, fetal anaemia, and fetal sex, with participating centre as a random intercept. The solid line represents the adjusted odds ratio and the shaded area its 95% confidence interval (CI). Gestational age at birth was associated with severe brain injury overall (p=0.009), without evidence of non-linearity (p=0.521).

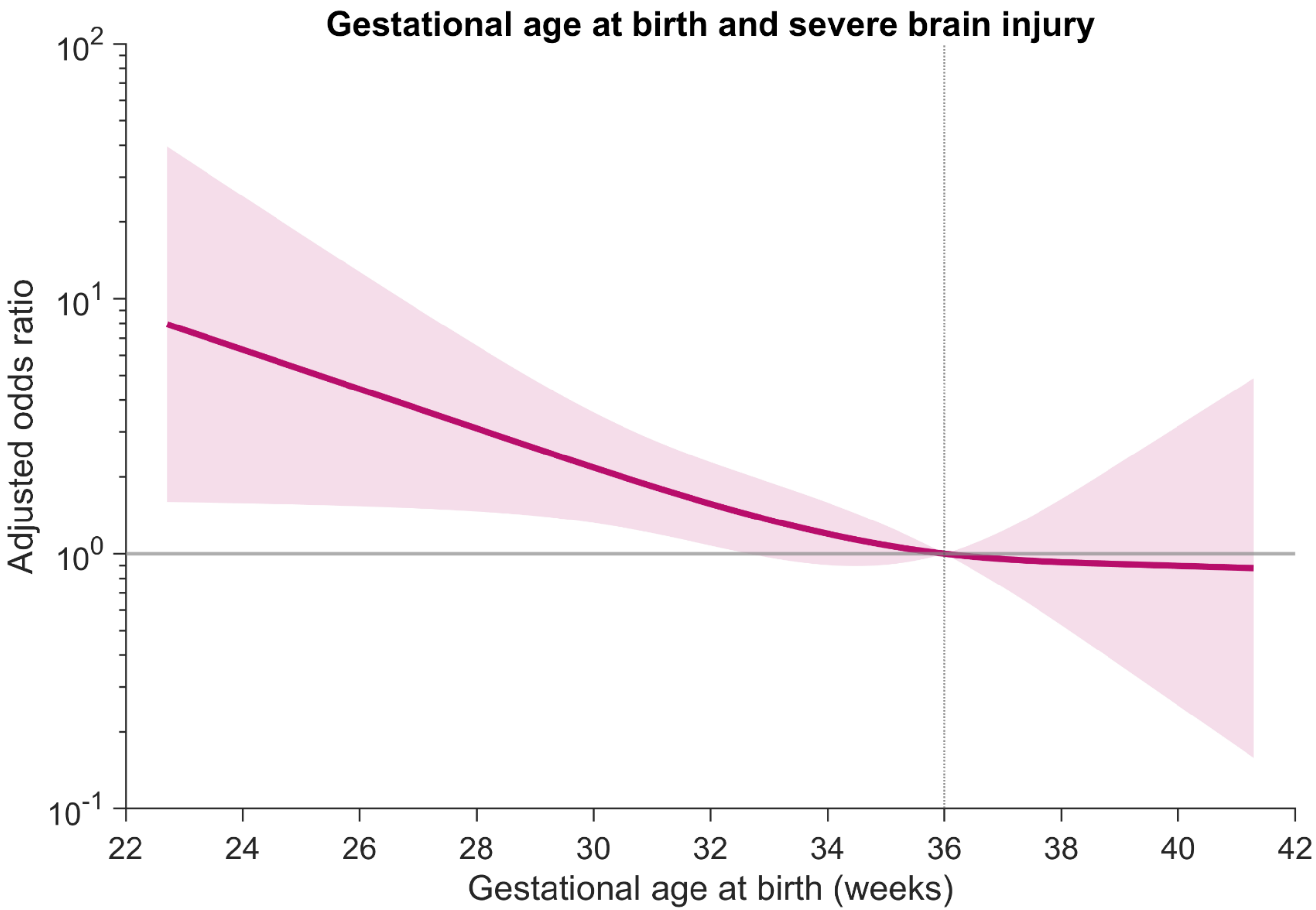

Supplementary Tables

Supplementary Table 1. Centre-specific recruitment periods and referral characteristics

Participating centre	Country	Recruitment period	Referral setting
BCNatal, Hospital Clinic and Hospital Sant Joan de Deu	Spain	2019-2025	National referral centre
Children's Hospital 'Vittore Buzzi'	Italy	2000-2025	National referral centre
Hospital Italiano de Buenos Aires	Argentina	2014-2024	National referral centre
Karolinska University Hospital	Sweden	2006-2025	National referral centre
Liverpool Women's Hospital & St George's Hospital	United Kingdom	2000-2025	National referral centre
Mount Sinai Hospital & The Hospital for Sick Children	Canada	2010-2025	National referral centre
Ospedale Pediatrico Bambino Gesù	Italy	2020-2025	National referral centre
Sheba Medical Centre	Israel	2011-2024	National referral centre
Technical University of Munich	Germany	2015-2024	Regional referral centre
University Hospitals Leuven	Belgium	2006-2025	International referral centre
Willem-Alexander Children's Hospital	The Netherlands	2002-2025	National referral centre

Recruitment periods represent the period during which each centre retrospectively identified all eligible cases from its local clinical databases. National and regional referral centre classifications reflect the geographical referral of each participating centre.

Supplementary Table 2. Ethics approval and informed-consent requirements by participating centre

Participating centre	Ethics committee/IRB	Reference number	Ethics status	Informed consent
BCNatal, Hospital Clinic and Hospital Sant Joan de Deu	Institutional Ethics Committee, CEIm del Hospital Clínic i Provincial and CEIm Fundació Sant Joan de Déu; Barcelona	HCB/2025/0992 and PIC-216-25 (acta 24/2025)	Approved by the local ethics committee/IRB	Informed consent was waived by the ethics committee or competent authority
Children's Hospital 'Vittore Buzzi'	Comitato etico territoriale Lombardia 1	1699/23th dec 2025	Approved by the local ethics committee/IRB	Written informed consent was obtained
Hospital Italiano de Buenos Aires	Research Protocol Ethics Committee (CEPI) of the Hospital Italiano de Buenos Aires	7218	Approved by the ethics committee/IRB (CEPI)	Informed consent was waived by the ethics committee.
Karolinska University Hospital	Swedish Ethical Review Authority	Dnr 2025-00073-01	Swedish Ethical Review Authority approval on February 17th 2025	Informed consent was not required. Data were pseudonymised in accordance with institutional and GDPR requirements
Liverpool Women's Hospital & St George's Hospital	Ymchwil Iechyd a Gofal Cymru Health and Care Research Wales	24/HRA/5276	Approved	Not needed due to retrospective design
Mount Sinai Hospital & The Hospital for Sick Children	Sinai Health Research Ethics Board SickKids Research Ethics Board	REB# 0062 // 3873	Approved (exp. 1-May-2027)	Informed consent was waived
Ospedale Pediatrico Bambino Gesù	EC Bambino Gesù Hospital	23-3065	EC Bambino Gesù approval on April 16th 2025	Written informed consent was obtained.
Sheba Medical Centre	Institutional Review Board, Sheba Medical Center, Tel HaShomer	SMC1552-24	Approved by the local ethics committee/IRB	Informed consent was waived by the ethics committee. The study was a retrospective analysis of existing, de-identified clinical data, with no additional risk to patients.

Technical University of Munich	Ethics Committee Technical University of Munich	2025-341-S-NP	Approved by the local ethics committee/IRB	Existing written consent covered the use of the data for this research
University Hospitals Leuven	EC Research UZ/KU Leuven	S70004	EC Research UZ/KU Leuven approval on May 9th 2025	Informed consent was not required. Data were pseudonymised in accordance with institutional and GDPR requirements.
Willem-Alexander Children's Hospital	Non-WMO Committee, Division 3, Leiden University Medical Center	23-3065	Reviewed and deemed not subject to the Dutch Medical Research Involving Human Subjects Act (non-WMO)	Opt-out since June 2017. Consent was waived.

Supplementary Table 3. Centre-specific obstetric management after single fetal demise in monochorionic twin pregnancies

The study period spanned 2000–2025, during which imaging and management practices may have changed over time. The information presented in this table reflects the current guidelines and standard clinical practice at each participating institution.

Center	Admission after sFD	Fetal monitoring after sFD	Antenatal corticosteroids after sFD	Magnesium Sulphate after sFD	Indication for delivery	Planned gestational age at birth after sFD
BCNatal, Hospital Clinic and Hospital Sant Joan de Deu	Not routinely	Individualized ultrasounds follow up, and measurement of peak systolic velocity in middle cerebral artery	Not routinely	Not routinely	Suspicion of fetal compromise, otherwise individualized.	36-37 weeks if clinically stable, 34 weeks if fetal compromise is suspected
Children's Hospital 'Vittore Buzzi'	Not routinely	Ultrasound as soon as possible. If signs of fetal anemia or compromise are detected, daily surveillance is performed	Administered in case of threatened preterm birth between 23 and 34 weeks of gestation	Administered in case of imminent delivery before 32 weeks of gestation	Single fetal demise is not considered an indication for early delivery. Timing of delivery is individualized based on fetal wellbeing, overall clinical course, and parental preference.	Preferably between 39-40 weeks of gestation. Delivery not advised beyond 40 weeks of gestation
Hospital Italiano de Buenos Aires	No	Ultrasound approximately 3 days after demise. Further ultrasound follow-up is individualized, every week for the first 2-3 weeks and then every 2 weeks (depending on the clinical course and parental preference)	No (unless threatened preterm birth between 23 and 34 weeks of gestation)	No (except with imminent delivery up to 34 weeks)	Single fetal demise is not considered an indication for (early) delivery. Timing of delivery is individualized aiming for 36 weeks.	36-37 weeks
Karolinska University Hospital	Not routinely.	Depending on the gestational age	Not administered routinely, but in case of threatened preterm birth before 34 weeks of gestation	Not administered routinely, but in case of imminent preterm birth before 32 weeks	Fetal compromise requiring urgent delivery. Timing of delivery may be individualized based on fetal growth, the clinical course, and parental preferences.	Preferably between 37-40 weeks of gestation depending on clinical course.
Liverpool Women's Hospital & St George's Hospital	No	Depending on the gestational age	Depending on the gestational age	No	Fetal compromise requiring urgent delivery	36-37 weeks
Mount Sinai Hospital & The Hospital for Sick Children	No	Ultrasound ~1 week after single fetal demise. Ultrasound follow-up is individualized, every 2 weeks (depending on the clinical course and parental need for reassurance)	Not routinely	Not routinely	Single fetal demise is not considered an indication for (early) delivery. Routine obstetric indications for delivery.	37 weeks

Ospedale Pediatrico Bambino Gesù	No	Ultrasound within 24 hours, at 1 week if normal, and every 2 weeks thereafter.	Only in case of threatened preterm birth between 23+5 and 34 weeks of gestation	Only when delivery before 32 weeks of gestation was considered imminent	Only obstetrical complications	37-38 weeks
Sheba Medical Centre	Not routinely	Ultrasound follow-up is individualized, typically peak systolic velocity of middle cerebral artery measurement at diagnosis and 1 day after, then every 7–10 days	After 24 weeks of gestation	Not routinely administered. Only if delivery before 32 weeks is considered imminent.	Single fetal demise alone is not an indication for early delivery. Timing is individualized according to fetal growth, fetal well-being, and standard obstetric indications	Timing of delivery is individualized aiming for 36-37 weeks of gestation.
Technical University of Munich	Not routinely	Follow-up after 1 week if ultrasound at the time of demise diagnosis is normal. Thereafter, every 2 weeks if clinically stable.	Not routinely administered	Not routinely	Single fetal demise is not considered an indication for delivery. Usual obstetric indications.	36-37 weeks if clinically stable
University Hospitals Leuven	No	Yes, cardiotocography after 28 weeks of gestation; repeat ultrasound scan after 1-3 weeks.	No	No	In the event of fetal distress of the surviving co-twin in the viable period in consultation with parents	Elective birth 37–39, in case of monoamniotic twins with cord entanglement 32-33 weeks
Willem-Alexander Children’s Hospital	Not routinely	Ultrasound approximately 1 week after single fetal demise. Further ultrasound follow-up is individualized, every 2–3 weeks, depending on the clinical course and parental preference	Not administered routinely, but only in case of threatened preterm birth between 23+5 and 34 weeks of gestation	Not administered routinely, but only when delivery before 32 weeks of gestation was considered imminent	Fetal demise is not considered an indication for (early) delivery. Timing of delivery may be individualized based on fetal growth, the clinical course, and parental preferences.	Preferably between 38-40 weeks of gestation. Delivery not advised beyond 40 weeks of gestation

Supplementary Table 4. Centre-specific neuroimaging protocols after single fetal demise in monochorionic twin pregnancies

The study period spanned 2000–2025, during which imaging and management practices may have changed over time. The information presented in this table reflects the current guidelines and standard clinical practice at each participating institution. Abbreviations: DWI, diffusion weighted imaging; single fetal demise, sFD.

Center	Routine antenatal neurosonography after sFD	Routine fetal MRI after sFD	Postnatal cranial ultrasound protocol	Postnatal MRI protocol
BCNatal, Hospital Clinic and Hospital Sant Joan de Deu	Yes, approximately 4 weeks after sFD (around 28 to 30 weeks)	Yes, at 30 to 32 weeks, or before if fetal compromise is suspected	Not routinely performed. Only if prematurity, abnormal prenatal neuroimaging, neurological symptoms, or other clinical indications.	If abnormal prenatal/postnatal imaging or clinically indicated
Children's Hospital 'Vittore Buzzi'	Yes, approximately 2-3 weeks after sFD	Yes, after 2-3 weeks with a gestational age ≥ 21 weeks.	Routinely in all survivor neonates within 3 days after birth	If abnormal prenatal/postnatal imaging or clinically indicated.
Hospital Italiano de Buenos Aires	Yes, 3-4 weeks after sFD	Yes, 3-4 weeks after demise	Routinely in complicated monochorionic twins within 2-3 days after birth	If abnormal prenatal/postnatal imaging or clinically indicated
Karolinska University Hospital	Yes, 2-4 weeks after sFD	Not routinely, depending on gestational age and findings on neurosonography	Cranial ultrasound day 4-7, earlier day 0-3 if neurological symptoms. If preterm birth: Follow-up ultrasound at 4 weeks of age and full-term corrected age. If pathological exam: follow-up with weekly cranial ultrasound	If abnormal prenatal/postnatal imaging or clinically indicated. MRI routinely performed at full-term corrected age in all infants born extremely preterm <28 weeks gestation
Liverpool Women's Hospital & St George's Hospital	Yes, 4-6 weeks after sFD	Yes, 4-6 weeks after demise	Yes, routinely.	If the postcranial ultrasound shows abnormalities or concerns
Mount Sinai Hospital & The Hospital for Sick Children	Yes, 2 weeks after sFD	Yes, fetal MRI at 28-30 weeks if neurosonography normal	Only in cases abnormal prenatal brain imaging or if gestational age at birth <32 weeks or low birthweight <1500 gr	In case of abnormal prenatal brain imaging or abnormal postnatal head US.
Ospedale Pediatrico Bambino Gesù	Yes, 2-3 week after sFD	Yes, 2-3 weeks after sFD	Within 3 days after birth	If abnormal prenatal/postnatal imaging or clinically indicated
Sheba Medical Centre	Yes, ultrasound with Doppler studies and neurosonography every 2 weeks until delivery	Yes, DWI-MRI within 72h after sFD, fetal brain MRI at 32 or 3-4 weeks after sFD if there is any finding on US or DWI-MRI	Routinely after sFD, ideally within 3 days after birth	Not routinely, depending on prenatal/postnatal imaging or if clinically indicated
Technical University of Munich	Yes, 4 weeks after sFD	Yes, around 30 weeks of gestation	If abnormal prenatal imaging or clinically indicated cranial ultrasound within 24h. If gestational age at birth < 32 weeks or birthweight <1.500 grams cranial ultrasound at day 1, 3, 5 and 7 of life.	If abnormal prenatal/postnatal imaging or clinically indicated

University Hospitals Leuven	Yes, 3 weeks after sFD	Yes, DWI at time of diagnosis and if >20 weeks; 30 weeks of gestation (at least 3 weeks after sFD)	If abnormal prenatal/postnatal imaging or clinically indicated	If abnormal prenatal/postnatal imaging or clinically indicated
Willem-Alexander Children's Hospital	Yes, 3-4 weeks after sFD	Yes, 4 weeks after sFD	Routinely in complicated monochorionic twins ideally within 3 days after birth	If abnormal prenatal/postnatal imaging or clinically indicated

Supplementary Table 5. Obstetric characteristics, management and pregnancy outcomes of included and excluded pregnancies

**Characteristics**	Pregnancies with an initially surviving cotwin (n=445)	Excluded pregnancies (n=50)	Included pregnancies (n=395)	p-value
Obstetric characteristics				
Median GA at sFD (weeks, IQR) *	22.6 (19.0-27.0)	21.9 (17.9-26.2)	22.7 (19.3–27.1)	0.17
TTTS (n, %)	151 (34)	22 (44)	129 (33)	0.11
Median GA at diagnosis (weeks, IQR) †	21.0 (18.6–24.0)	21.4 (18.4–24.0)	21.0 (18.6–23.9)	0.71
Median Quintero stage at diagnosis (I–IV, IQR)+	II (I–III)	II–III (I–III)	I (I–III)	0.22
Amnioreduction (n, %)	26 (6)	5 (10)	21 (5)	0.18
sFGR (n, %)	104 (23)	10 (20)	94 (24)	0.55
Median GA at diagnosis (weeks, IQR) ‡	18.4 (16.4-21.0)	16.9 (15.3-19.0)	18.6 (16.5–21.1)	0.09
Gratacós type at diagnosis (%)				0.84
Type I	33 (32)	2 (20)	31 (33)	
Type II	29 (28)	3 (30)	26 (28)	
Type III	33 (32)	4 (40)	29 (31)	
Unknown	9 (9)	1 (10)	8 (9)	
Monoamnionicity (n, %)	36 (8)	2 (4)	34 (9)	0.41
Post-sFD assessment and management				
MCA-PSV in survivor after sFD (MoM, IQR) §	1.3 (1.0-1.7)	1.1 (0.8-1.4)	1.3 (1.1–1.7)	0.01
Fetal anaemia	111/271 (41)	3/19 (16)	108/252 (43)	0.02
IUT	48/445 (11)	1/50 (2)	47/395 (12)	0.03
Prenatal glucocorticoids of infants born <32 weeks of gestation (n/N, %)	35/48 (73)	2/3 (67)	33/45 (73)	1.00
Caesarean delivery (n/N, %)	136/339 (40)	5/17 (29)	131/322 (41)	0.36
Pregnancy outcomes				
dFD (n, %)	66 (15)	28 (56)	38 (10)	<0.001
Median GA at diagnosis (weeks, IQR)¶	21.3 (18.6–26.0)	20.0 (17.9–23.7)	22.9 (19.0–26.5)	0.09
TOP (n, %)	39 (9)	5 (10)	34 (9)	0.79
Median GA at TOP (weeks, IQR)	25.0 (21.2–28.6)	23.3 (22.1–25.0)	25.6 (20.8–28.9)	0.92

Abbreviations: dFD, double fetal demise; GA, gestational age; IQR, interquartile range; IUT, intrauterine transfusion; MCA-PSV, middle cerebral artery peak systolic velocity; MoM, multiples of the median; sFD, single fetal demise; sFGR, selective fetal growth restriction; TOP, termination of pregnancy; TTTS, twin–twin

transfusion syndrome. Data are presented as n (%) or median (IQR). Denominators vary per variable due to missing data and are indicated in the table as n/N (%) or with footnotes.

*GA at sFD was available in 440/445 (99%) pregnancies.
† GA at TTTS diagnosis was available in 145/151 (96%) TTTS pregnancies.
+ Quintero stage was available in 96/151 (64%) TTTS pregnancies
‡ GA at sFGR diagnosis was available in 102/104 (98%) sFGR pregnancies.
§ MCA-PSV after sFD was available in 270/445 (61%) pregnancies.
¶GA at dFD was available in 63/66 (95%) dFD cases.

Supplementary Table 6. Neonatal characteristics and short-term outcome of liveborn infants after single fetal demise

**Neonatal characteristics of liveborn infants**				
Characteristics	Total (n=323)	Severe Brain injury (n=45)	No severe brain injury (n=278)	p-value
Median GA at birth (weeks, IQR) *	36.14 (32.50–38.00)	34.22 (28.78–36.54)	36.43 (33.00–38.07)	<0.001
Median interval between GA at sFD and birth of liveborn survivor (weeks, IQR) †	11.57 (3.57–17.86)	3.36 (0.14–9.54)	12.71 (4.86–18.14)	<0.001
Birthweight (grams, IQR) ‡	2420 (1700–2995)	2260 (1260–2562)	2500 (1797–3000)	0.004
Female (n/N, %)	143/268 (53)	20/37 (54)	123/231 (53)	0.93
Apgar score at 5 minutes (IQR)§	9 (8–10)	7 (5–9)	9 (9–10)	<0.001
SGA (n/N, %)	53/263 (20)	4/36 (11)	49/227 (22)	0.15
Hemoglobin level at birth (g/dL, IQR) ¶	15.2 (12.2–17.6)	11.7 (7.8–15.4)	16.1 (12.9–17.8)	<0.001
Blood transfusion at birth (n/N, %)	25/256 (10)	13/35 (37)	12/221 (5)	<0.001
Use of inotropics (n/N, %)	20/256 (8)	8/36 (22)	12/220 (6)	0.003
Neonatal death (n, %)	16/323 (5)	9/45 (20)	7/278 (3)	<0.001
Neonatal comorbidity				
Severe neonatal morbidity	72/262 (28)	20/36 (56)	52/226 (23)	<0.001
Perinatal asphyxia (n/N, %)	15/262 (6)	9/36 (25)	6/226 (3)	<0.001
Respiratory distress syndrome (n/N, %)	65/262 (25)	19/36 (53)	46/226 (20)	<0.001
Necrotizing enterocolitis (n/N, %)	3/262 (1)	0/36 (0)	3/226 (1)	1.00
Early-onset sepsis (n/N, %)	10/262 (4)	1/36 (3)	9/226 (4)	1.00
Retinopathy of prematurity (n/N, %)	7/262 (3)	3/36 (8)	4/226 (2)	0.06
Bronchopulmonary dysplasia (n/N, %)	9/262 (3)	1/36 (3)	8/226 (4)	1.00

Abbreviations: GA, gestational age; IQR, interquartile range; SD, standard deviation; SGA, small for gestational age.

Data are presented as n (%) or median (IQR). Denominators vary per variable due to missing data and are indicated in the table as n/N (%) or with footnotes.
* Median GA at birth was available for 88% (285/323) of liveborn infants.
† Median interval between GA at sFD and live birth of the survivor was available for 88% (285/323) of liveborn infants.
‡ Birthweight was available for 83% (267/323) of liveborn infants
§ Apgar scores at 5 minutes were available for 67% (215/323) of liveborn infants.
¶ Hemoglobin levels at birth were available for 34% (110/323) of liveborn infants.

Supplementary Table 7. Documented postnatal neuroimaging by gestational age at birth and participating centre

Characteristic	Liveborn infants, n	Documented postnatal neuroimaging, n/N (%)	p-value
Overall	323	160/323 (49.5)	
Gestational age at birth*			<0.0001†
<28 weeks	24	16/24 (66.7)‡	
28–31+6 weeks	37	34/37 (91.9)	
32–36+6 weeks	106	68/106 (64.2)	
≥37 weeks	118	40/118 (33.9)	
Participating centre			<0.0001§
Centre A	12	9/12 (75.0)	
Centre B	41	14/41 (34.1)	
Centre C	61	10/61 (16.4)	
Centre D	23	8/23 (34.8)	
Centre E	8	3/8 (37.5)	
Centre F	19	16/19 (84.2)	
Centre G	74	47/74 (63.5)	
Centre H	6	5/6 (83.3)	
Centre I	57	38/57 (66.7)	
Centre J	3	3/3 (100.0)	
Centre K	19	7/19 (36.8)	

*Data are n/N (%). Participating centres were anonymised using alphabetical labels. * Gestational age at birth was available for 88.2% (285/323) of liveborn infants. † Pearson χ^2 test. ‡ Four of the eight infants born before 28 weeks without documented postnatal neuroimaging died before cranial ultrasound could be performed. § Fisher exact test.*

Supplementary Table 8. Sensitivity analyses for incomplete postnatal neuroimaging

Analysis	Severe brain injury
All liveborn infants	45/323 (13.9%)
With documented postnatal neuroimaging	32/160 (20.0%)
With antenatal and postnatal neuroimaging	30/149 (20.1%)
Assumed prevalence of undetected severe brain injury among 150 infants without neuroimaging†	Estimated prevalence among 323 liveborn infants
0%	13.9%
5%	16.3%
10%	18.6%
15%	20.9%

†Of 323 liveborn infants, 160 had documented postnatal neuroimaging and 163 did not. Severe injury was detected antenatally in 8% (13/163) infants and was included in the 45 observed cases. The remaining 150 infants had no antenatally detected severe injury, but additional brain injury could not be excluded without postnatal imaging. Estimated prevalence was calculated as [45+(150×p)]/323×100, where 45 represents the observed cases of severe brain injury, 150 the infants without postnatal neuroimaging or antenatally detected severe injury, and p the assumed proportion with undetected injury.

Supplementary Table 9. Sensitivity analyses of the multivariable model for severe brain injury following single fetal demise

Panel A. Primary, complete-case, and antenatal-origin analyses

Factor	Primary model, aOR (95% CI, 395/72)	p value	Complete cases, aOR (95% CI, 188/32)	p value	Antenatal-origin analysis, aOR (95% CI, 382/59)	p value
GA at sFD, per week later	1.09 (1.03–1.14)	0.001	1.06 (0.98–1.14)	0.157	1.06 (1.00–1.12)	0.034
Twin-to-twin Transfusion	0.56 (0.30–1.05)	0.069	0.89 (0.38–2.09)	0.784	0.49 (0.24–0.98)	0.044
Selective fetal growth restriction	0.86 (0.48–1.56)	0.628	0.95 (0.41–2.21)	0.904	0.94 (0.50–1.77)	0.855
Monoamnionicity	0.71 (0.25–2.00)	0.516	0.68 (0.14–3.38)	0.636	0.48 (0.13–1.75)	0.268
Fetal anaemia	2.29 (1.24–4.25)	0.009	2.03 (0.92–4.50)	0.082	2.47 (1.27–4.81)	0.008
Male sex	0.79 (0.39–1.59)	0.509	0.91 (0.41–2.00)	0.810	0.66 (0.29–1.53)	0.336

Data are adjusted odds ratios (aORs) with 95% confidence intervals (CIs). All analyses used mixed-effects logistic regression with participating centre as a random intercept and included GA at sFD, TTTS, sFGR, monoamnionicity, fetal anaemia, and fetal sex. The primary and antenatal-origin analyses used multiple imputation by chained equations with 50 imputed datasets and 20 iterations. The complete-case analysis included the 188 pregnancies with complete data for all model variables. The antenatal-origin analysis retained the 323 pregnancies without severe brain injury and the 59 pregnancies with severe brain injury of antenatal origin; four perinatal, seven postnatal, and two indeterminate severe injuries were excluded.

Panel B. Missing-not-at-random sensitivity analysis for fetal anaemia

Assumption for originally missing fetal-anaemia values	Delta-OR	Mean imputed fetal anaemia among missing values	Fetal anaemia aOR (95% CI)	p value
Lower odds of fetal anaemia	0.5	25.5%	2.43 (1.29–4.58)	0.006
Missing at random	1.0	39.2%	2.29 (1.24–4.25)	0.009
Higher odds of fetal anaemia	2.0	54.5%	2.06 (1.11–3.82)	0.022

In panel B, delta adjustment was applied only to originally missing fetal-anaemia values; observed values were retained unchanged. Delta-ORs of 0.5 and 2.0 respectively halve and double the conditional odds of fetal anaemia relative to the missing-at-random imputation model. Each scenario included 395 pregnancies and 72 severe brain injury events, used 50 imputations, and applied the same primary mixed-effects model. Percentages represent the mean proportion imputed with fetal anaemia across the 50 imputed datasets.

Study Protocol

CITRUS Study protocol

CITRUS-study

(Brain Injury In Monochorionic Twins After Intrauterine Demise: An International Multi-Center Retrospective Study)

Research Protocol

PROTOCOL TITLE 'CITRUS-study: *Brain Injury in Mono**C**horion**I**c **T**wins Afte**R** Intra**U**terine demi**S**e: a international multi-center retrospective study*

**Short title**	CITRUS-study
**EudraCT number**	Not applicable
**Version**	3.0
**Date**	14-06-2024
**Coordinating investigator/project leader**	M. (Mathies) Rondagh, PhD student<br>Department of Neonatology, the Netherlands,<br>Leiden University Medical Center (LUMC)<br>Albinusdreef 2, 2333ZA Leiden, the Netherlands<br>m.rondagh@lumc.nl<br>Tel: +31 (0)71-5262965
**Principal investigator(s)**	prof. dr. E. (Enrico) Lopriore<br>Department of Neonatology<br>Leiden University Medical Center<br>e.lopriore@lumc.nl<br>Tel: +31 (0)71-5262965

**Local Investigators**	Personal contact details have been removed. The scientific content has not been modified.
**Sponsor**	Leiden University Medical Center<br>Albinusdreef 2<br>2333 ZA Leiden, The<br>Netherlands
**Subsidizing parties**	• European Society for Paediatric Research (ESPR)<br>• Leids Universitair Fonds (LUF) and the Gratama Stichting (2023-10/W233029-2-GSL)<br>• Strong Babies (SB-JO-2023-15)<br>• Honours College Leids Universitair Medisch Centrum (240426)<br>• Janivo Stichting<br>• Gisela Thier Fonds<br>• Raynor Foundation<br>• Vaillant Stichting

PROTOCOL SIGNATURE SHEET

Personal contact details and signatures have been removed. The scientific content has not been modified.

PROTOCOL AGREEMENT PAGE

I agree to conduct the retrospective study in accordance with the current protocol and comply with its requirements, subject to ethical and safety considerations.

Institute ……………………………………

Local Investigator Name ……………………………………

Local Investigator Signature ……………………………………

Date ……………………………………

INTRODUCTION AND RATIONALE

Monochorionic (MC) twin pregnancies are at risk of several complications arising from sharing a single placenta, including twin-twin transfusion syndrome (TTTS), twin anaemia polycythaemia sequence (TAPS), and selective fetal growth restriction (sFGR).[1] In TTTS the presence of placental vascular anastomoses causes an imbalanced blood exchange between the twins, resulting in polyuria and polyhydramnios in the recipient twin and oliguria and oligohydramnios in the donor twin.[1] TAPS is a rare condition in which persistent transfusion results in anaemia in the donor twin and polycythaemia in the recipient twin.[1] Lastly, sFGR is a complication characterized by unequal placental sharing, resulting in significant intertwin growth discordance. So, MC twin pregnancies are considered high risk pregnancies with significant perinatal mortality rates, including single or double intrauterine fetal demise (IUFD).[1] In case of single fetal demise (sIUFD), the surviving co-twin is known to be at risk of brain injury which can have consequences for neurodevelopmental outcomes in the future.[2-8]

The commonly held hypothesis regarding brain injury after sIUFD in the surviving co-twin is that it arises due to the abrupt exsanguination of the surviving co-twin into the low-pressure circulation of the deceased co-twin.[9,10] This immediate blood loss initiates acute hemodynamic alterations, marked by hypovolemia and anaemia, which subsequently leads to hypoxic-ischemic injury and multiorgan failure.[9,10] Hypothetically, the presence of large arterio-arterial or veno-venous anastomoses may allow a more rapid and massive exsanguination owing to the low resistance of these anastomoses.[9] Another possible mechanism that could lead to brain injury in survivors after sIUFD is thrombus formation within the vascular compartment of the demised fetus. This thrombus could pass to the surviving twin because of the persistent flow in the placental anastomotic vessels. These emboli, upon reaching the circulation of the surviving fetus, may become lodged within the cerebral blood vessels, thereby potentially leading to focal ischemic lesions.[11] Previous research reported that 10-26% of surviving co-twins after a sIUFD were diagnosed with severe brain injury. Most brain injuries were identified postnatally (18%) and a smaller proportion before birth (8%) using fetal neurosonography or neonatal cerebral ultrasonography (CUS) and/or magnetic resonance imaging (MRI). [9,11] However, knowledge of the exact mechanisms and potential risk factors leading to different types of brain injury after sIUFD remains limited. There is also a lack of data on the postnatal clinical outcomes of surviving co-twins.

Therefore, this study aimed to describe the incidence, type, and severity of brain injury in surviving co-twins after sIUFD in MC twin pregnancies and to characterize the neurodevelopmental outcomes of surviving co-twins in relation to identified brain injuries. The secondary aim was to identify the potential risk factors for brain injury based on obstetric and placental characteristics.

OBJECTIVES

Primary Objectives

1. To describe the incidence, type, and severity of brain injury in surviving co-twins after single fetal intrauterine demise.

2. To characterize the neurodevelopmental outcomes of surviving co-twins in relation to identified brain injuries.

Secondary Objectives

1. To identify potential risk factors for brain injury based on obstetric, patient, and placental characteristics.

2. To determine the residual risk of neurodevelopmental impairment following normal fetal and/or neonatal neuroimaging.

STUDY DESIGN

Study design

We will perform an international multicenter retrospective cohort study. Enrollment will take place by contacting international expertise centers and asking for their participation in the CITRUS study (Figure 1). We will send the latest version of the protocol for submission to the institutional review boards of participating centers. Following the approval of the review board, our privacy officer will forward a data sharing agreement to all parties involved. After the agreement is signed, local investigators will receive access to our Castor Database to upload the data. Cases will be pseudo-anonymized, and data will be collected in a Castor database (a protected online database).

We will arrange an agreement session for all neuroimaging modalities, including fetal neurosonography, fetal MRI, neonatal cerebral ultrasound, and neonatal MRI. During these sessions, all centers will first individually score various cases, which will then be discussed in a plenary session. This approach ensures that consistent definitions are used during data collection.

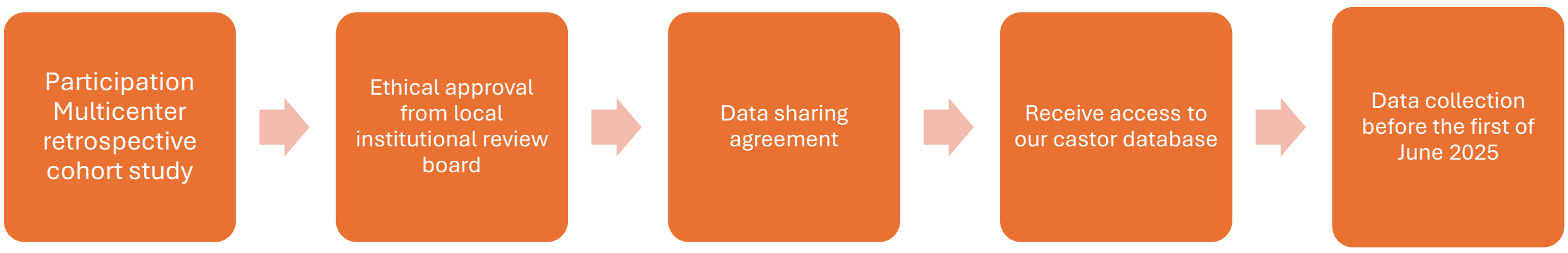

Figure 1. Study Design

Duration

Data will be collected and added to the Castor database before the first of June 2025 (if necessary and upon agreement, an extension may be granted).

STUDY POPULATION

Population (base)

Monochorionic twin pregnancies with sIUFD or double IUFD (dIUFD) in one of the participating hospitals or affiliated hospitals between January 2000 and January 2025 are eligible for inclusion.

Inclusion criteria

To be eligible to participate in this study, a subject must meet all the following criteria.

1. MC pregnancies complicated by either sIUFD or dIUFD, which occur ≥12 weeks of gestation (crown-rump length at least of one of the twins at least >10 weeks of gestation).

Exclusion criteria

Potential subjects who meet any of the following criteria will be excluded from participation in this study:

1. sIUFD or dIUFD occurring after laser treatment for TTTS
2. Higher-order pregnancies
3. TAPS
4. TRAP
5. Selective feticide
6. Major congenital anomalies or malformations in one or both twins

METHODS

Main study endpoint

Incidence, type and severity of brain injury in surviving cotwin

One of the primary objectives of this study is the incidence, type, and severity of brain injury in the surviving co-twin. We will record the presence and characteristics of fetal and/or neonatal brain injury detected by antenatal and/or postnatal neuroimaging. We will also describe whether fetal neurosonography, fetal MRI, neonatal brain ultrasound, and neonatal MRI were performed, including GA or post-conceptional age at which neuroimaging was performed, and describe the type, location and severity of brain injury. Baseline characteristics of neuroimaging will be collected, including the MRI-sequences, magnetic field strength, ultrasound windows and planes, probes and frequency of the probes.

Brain injury will be categorized as either diffuse or focal injury and divided further into subgroups, as presented in Table 1 according to Spruijt et al.[12] Diffuse brain injury will be divided into white matter injury (cystic periventricular leukomalacia (PVL) according to de Vries, or punctate white matter lesions (PWML, unilateral or bilateral)) multicystic or generalized encephalomalacia, migration or gyration disorders, ventriculomegaly and symmetrical volume loss. Ventriculomegaly (VM) will be defined as an atrial diameter ≥ 10 mm on fetal US as well as fetal and postnatal MRI or a ventricular index > p97 on neonatal cUS.[12,13] Focal brain injury will be defined as an arterial ischemic infarction, intraventricular hemorrhage (IVH), intraventricular hemorrhage with periventricular hemorrhagic infarction (PVHI), or cerebellar or cerebral parenchymal hemorrhage. VM will be termed post-hemorrhagic ventricular dilatation (PHVD) if preceded by intraventricular hemorrhage (IVH) ≥ grade 2, according to Volpe [14]. Cerebellar hemorrhage (CBH) detected with postnatal cUS or MRI will be subdivided as punctate(< 5 mm) , or limited/ massive.[12] Infarction will be diagnosed when cUS showed an area of increased echogenicity within an arterial territory or MRI/diffusion weighed imaging (DWI) showed typical signal abnormalities in the acute stage, and/or subsequent tissue loss.

Table 1. Brain injury categories and groups.

**Category/Group**	**Group**	**Injury**
Diffuse	1	Cystic periventricular leukomalacia or punctate white matter lesions
	2	Multicystic or generalized encephalomalacia
	3	Migration or gyration disorders
	4	Ventriculomegaly and/or severe volume loss
Focal	5	Infarction
	6	Intraventricular hemorrhage (with or without post-hemorrhagic ventricular dilatation)
	7	Intraventricular hemorrhage with periventricular hemorrhagic infarction
	8	Cerebellar or cerebral parenchymal hemorrhage

Severe brain injury will be defined as the presence of IVH grade III and IVH with PVHI, IVH with PHVD, cystic PVL or multicystic encephalomalacia, a cerebellar or intraparenchymal hemorrhage ≥ 5 mm (so excluding punctate hemorrhage), arterial ischemic infarction, or other severe brain injuries associated with an adverse neurological outcome.

Neurodevelopmental outcome related to brain Injury

Another primary objective is the to characterize the neurodevelopmental outcomes of surviving co-twins in relation to identified brain injuries. Cognitive, neurological and/or motor development will be determined in case it was assessed using age appropriate tests. The used test will be described, including the age of assessment and the results.

In our institution, if available we performed, the Bayley Scales of Infant and Toddler Development third edition (Bayley-III-NL) for children aged two to three years. In children aged three to six years, the Wechsler Preschool and Primary Scale of Intelligence third edition (WPPSI-III-NL) was performed. When children are seven years or older, the Wechsler intelligence Scale for Children fifth edition (WISC-V-NL) was performed. Regarding motor development, the Bayley-III-NL was used for children aged two to three years to generate motor test scores. For children aged two to seventeen years the results of a neurological exam to identify the presence and severity of CP was classified according to the Gross Motor Function Classification System (GMFCS). Also, the Movement Assessment Battery for Children (M-ABC, 2nd Edition) was used to score motor function. In case the results of the ages and stages questionnaire (ASQ) or the Parent Report of Children's Abilities (PARCA) are available this will be collected. [15]

Information about vision and hearing impairments will be collected. Mild visual impairment is defined as requiring treatment by an ophthalmologist, strabismus, or needing a correction of up to +/- 3 dioptres. Severe visual impairment is defined as blindness or being partially sighted. Mild hearing impairment is defined as hearing loss up to 30 dB. Severe hearing impairment includes bilateral deafness, treatment in an audiological center, neurosensory hearing loss, or hearing loss requiring amplification.

The incidence of mild and severe neurodevelopmental impairment (NDI) will be determined. Mild NDI was diagnosed in patients with at least one of the following conditions: CP stage one, mild cognitive/motor delay (scores < 85), mild visual and/or hearing impairment, and/or M-ABC p<15. For severe NDI at least one of the following conditions needs to be present: CP ≥ GMFCS-II, severe cognitive and/or motor delay (scores < 70), severe visual and/or hearing impairment and/or or M-ABC p<5.

Secondary study endpoints

Obstetric characteristics

Data will be collected on sIUFD and dIUFD, including the GA at diagnosis and the (suspected) cause (TTTS, sFGR, spontaneous, or free text). The following obstetric parameters will be recorded: gravidity, parity, prenatal glucocorticoid administration, mode of delivery, reason for delivery (spontaneous or elective), and amnionicity (monoamniotic or diamniotic). In addition, the use of intrauterine transfusion (IUT) will be collected, including fetal haemoglobin levels before IUT (g/dL) and after IUT. Other fetal neurosonography characteristics will be collected, including the peak systolic velocity (PSV) Doppler measurement of the fetal middle cerebral artery (MCA) of the surviving co-twin, and the last measured EFW for both co-twins (including GA). The number of terminations of pregnancy (TOP) will be collected.

sFGR is defined as an EFW or birthweight discordance of ≥20%. Antenatal UA Doppler classification will be collected according to Gratacós. [16] TTTS is defined as antenatal ultrasound criteria with oligohydramnios with the deepest vertical pocket of 2 cm or less in the twin sac of one fetus and polyhydramnios with the deepest vertical pocket of 8 cm or greater before 20 weeks of gestation or 10 cm or greater after 20 weeks of gestation in the twin sac of the other fetus. Data will be collected about the procedures: amnioreduction (number of procedures). TTTS stage will be described according to Quintero et al. [17]

Placental characteristics

Pictures of the placenta, *when available*, can be uploaded in the castor database (only in case placentas were dye injected ≤ 2 weeks after sIUFD or dIUFD. These pictures should include a scale (in mm or cm). We will score the number of arterio-arterial (AA), venovenous (VV) and arterio-venous (AV) anastomoses and in case of AV anastomoses the direction of the blood flow: arteriovenous from donor to recipient (AVDR) or arteriovenous from recipient to donor (AVRD). We will measure the diameter of each AA and VV anastomosis at the level of the vascular equator in millimetres. The total AA or VV diameter will be calculated in cases in which the placenta possessed multiple AA or VV anastomoses by adding the subsequent diameters. The total number of anastomoses will be calculated by adding up all AA, VV, AV,

VA anastomoses.[18] The time (in days) between dye injection of the placenta and the single fetal demise will be calculated. The proportion of fetuses with a central, marginal (<1cm from the margin of the placenta) or velamentous umbilical cord insertion will be documented for the smaller and larger twins.[19] The placental share of the smaller twin will be documented. The placental territory ratio will be calculated by dividing the placental territory of the larger twin by that of the smaller one. The placental share discordance will be calculated as larger placental share−smaller placental share/larger placental share×100.[18]

Neonatal outcomes
The following neonatal parameters, when available will be collected from the medical records: GA at birth; birthweight, APGAR scores at 5 and 10 min; sex; Severe neonatal morbidity will be defined as the presence of at least one of the following conditions: perinatal asphyxia (5-minute Apgar score ≤5, need for resuscitation, mechanical ventilation for ≥10 minutes after birth, or cord or early neonatal blood pH <7.0, base excess <−16 mmol/L, or lactate >10.0 mmol/L within 1 hour after birth); respiratory distress syndrome (RDS) requiring mechanical ventilation; patent ductus arteriosus (PDA) requiring medical treatment or surgical closure; necrotizing enterocolitis (NEC) ≥ grade two [20]; proven neonatal sepsis defined as a clinically ill neonate with a positive blood culture; retinopathy of prematurity (ROP) staged according to ICROP [21]; bronchopulmonary dysplasia (BPD), defined as the need for supplemental oxygen for at least 28 days after birth, severity graded according to the respiratory support required at 36 postmenstrual weeks [22]. Neonatal death within 28 days after birth will be reported.

Study procedures
Data will be collected under the supervision of the local investigator. We will provide online data-entry training opportunities. Data will be collected from the hospital's written or electronic patient record files and imaging reports. The study data will be entered into the electronic data capture tool Castor. Study sites will have the opportunity to choose a preferred start date for data collection, aiming to start within 4 months after ethical approval by their local institutional review board and signing of the data sharing agreement (DSA). All participating local investigators will receive access to our castor database. The local investigators cannot access data from the LUMC or other participating centers in the castor database. All data were coded and stored in Datasafes and Castor, which are exclusively accessible to the coordinating investigator. ProMISe safeguards the key to uncoding the local data. The dataset automatically generates backups twice a day.

Overview of the study parameters, assessment tools, and definitions.

TYPE	STUDY PARAMETER	ASSESSMENT TOOL/DEFINITION (1/4)
**Main study parameters**	GA at diagnosis of IUFD	• Gestational age at the diagnosis of sIUFD<br>• Gestational age at the diagnosis of dIUFD (if applicable)<br>• Time in days between diagnosis of IUFD and fetal brain abnormalities.
	Fetal neurosonography	• Performed (0= no, 1 yes)<br>• Brain abnormalities detected (0= no, 1 yes)<br>• Gestational age at which brain abnormality detected (week + days)<br>• Sequential fetal neurosonography performed after injury (n)<br>• Type of abnormality (options provided in *Type, and free text)*<br>• Severity of abnormality (normal, mild, or severe)
	Fetal MRI	• Performed (0= no, 1 yes)<br>• Brain abnormalities detected (0= no, 1 yes)<br>• Gestational age at which brain abnormality detected (week + days)<br>• Type of abnormality (options provided in *Type, free text)*<br>• Severity of abnormality (normal, mild, or severe)
	Neonatal Cerebral Ultrasound	• Performed (0= no, 1 yes)<br>• Brain abnormalities detected (0= no, 1 yes)<br>• Postconceptional age at which abnormality detected (week + days)<br>• Type of abnormality (options provided in *Type, free text)*<br>• Severity of abnormality (normal, mild, or severe)
	Neonatal MRI (including DWI)	• Performed (0= no, 1 yes)<br>• Brain abnormalities detected (0= no, 1 yes)<br>• Postconceptional age at which abnormality detected (week + days)<br>• Type of abnormality (options provided in *Type, free text)*<br>• Severity of abnormality (normal, mild, or severe)
	Type of injury	Type of brain injury was categorized as either diffuse or focal injury and divided further into subgroups, as presented in Table 1 according to Spruijt et al.[12]
	Severe brain injury	Severe brain injury was defined as [10]:<br>• Intraventricular hemorrhage grade III and/or IVH with PVHI of PHVD<br>• Periventricular leukomalacia ≥ grade II<br>• Porencephalic cysts following PVHI<br>• Arterial ischemic infarction<br>• Intraparenchymal hemorrhage ≥ 5 mm<br>• Ventricular dilation ≥ 97th percentile<br>• Basal ganglia, thalamic and/or cortical injury<br>• Cerebellar injury > 4 mm<br>• Multicystic or generalized encephalomalacia
	Cystic periventricular leukomalacia (PVL)	PVL was defined according to de Vries et al. [13]
	Migration or gyration disorders	Migration or gyration disorders are characterized by: [15]<br>• Focal cortical dysplasia<br>• Gray matter heterotopia<br>• Lissencephaly<br>• Schizencephaly
	Encephalomalacia	Very extensive and diffuse brain injury reflecting an end result of liquefactive necrosis of brain parenchyma following insult, usually occurring after severe cerebral ischemia, cerebral infection, hemorrhage, traumatic brain injury, surgery or other insults
	Ventriculomegaly and/or symmetrical volume loss	Ventriculomegaly (VM) was defined as an atrial diameter ≥ 10 mm on fetal US (mild: 10-12mm; moderate 12-15mm; severe: >15mm) as well as fetal MRI, or a ventricular index > p97 (severe >p97 +4 mm) on neonatal cUS or neonatal MRI.

	Infarction	Infarction was diagnosed when cUS shows an area of increased echogenicity within an arterial territory, or MRI/DWI showed typical signal abnormalities in the acute stage, and/or corresponding tissue loss in the subacute stage
	Intraventricular hemorrhage (IVH)	Degree of hemorrhage is graded I through IV:<br>• Grade I – hemorrhage limited to Germinal matrix<br>• Grade II – IVH without ventricular dilatation<br>• Grade III - IVH occupying > 50% of the ventricle with acute ventricular dilatation<br>• Grade IV – IVH with periventricular hemorrhagic infarction [13]
	Cerebellar or cerebral parenchymal hemorrhage	Cerebellar hemorrhage (CBH) detected with postnatal cUS, or MRI was subdivided as punctate (< 5 mm), vs limited, or massive.

TYPE	STUDY PARAMETER	ASSESSMENT TOOL/DEFINITION (2/4)
**Secondary study characteristics (Obstetric characteristics)**	Single intrauterine demise (sIUFD)	sIUFD is defined as one of the co-twins dies during gestation.
	Double intrauterine demise (dIUFD)	dIUFD is defined as both co-twins dies during gestation.
	Gestational age of the diagnosing sIUFD	Gestational age at which the sIUFD was first detected
	Cause of sIUFD	Cause of sIUFD, in case there is a valid cause possible (e.g., TTTS, sFGR, spontaneous, etc.)
	Intrauterine transfusion	Intrauterine transfusion
	Hemoglobin level at intrauterine transfusion (IUT)	Hemoglobin level at IUT (number g/dL)
	Selective fetal growth restriction (sFGR)	sFGR is defined as an EFW or birthweight discordance of ≥20% [23]
	Gratacós score	Gratacós score defined according to Gratacos et al.[16], with the most prevalent type during pregnancy being chosen<br>• Type 1: Positive end-diastolic flow<br>• Type 2: Persistent absent or reversed end-diastolic flow<br>• Type 3: Intermittent absent or reversed end-diastolic flow
	TTTS	TTTS was is defined according to the following antenatal ultrasound criteria [19]:<br>• Monochorionicity established by absence of a “twin peak” sign and presence of a thin dividing membrane<br>• Oligohydramnios (deepest vertical pocket of 2 cm or less) in the twin sac of 1 fetus<br>• Polyhydramnios (deepest vertical pocket of 8 cm or greater before 20 weeks of gestation or 10 cm or greater after 20 weeks of gestation) in the twin sac of the other fetus.
	Quintero Stage	Defined as the following stages [17]:<br>• Stage I: bladder in the donor twin still visible<br>• Stage II, bladder in the donor twin no longer visible, no absent/reverse end-diastolic velocity in the umbilical artery, reverse flow in the ductus venosus, or pulsatile flow in the umbilical vein<br>• Stage III, absent/reverse end-diastolic velocity in the umbilical artery, reverse flow in the ductus venosus, or pulsatile flow in the umbilical vein<br>• stage IV, hydrops<br>• stage V, demise of one or both twins
	Amnio drainage	Amnio drainage under continuous ultrasound guidance to remove amniotic fluid.[24]
	Monoamniocity	Rare form of monozygous twins in which both fetuses occupy the same sac. [25]
	Delivery mode	Cesarean section or vaginal delivery.

TYPE	STUDY PARAMETER	ASSESSMENT TOOL/DEFINITION (3/4)
**Secondary Study characteristics (Placental characteristics)**	Placenta injected	Injection of the placenta with colored dye [18]
	Arterioarterial (AA) anastomoses	Number of arterio-arterial anastomoses. [18]
	Diameter of the AA anastomosis	Total diameter of AA anastomosis. [18]
	Venovenous (VV) anastomoses	Number of vein to vein anastomoses. [18]
	Diameter of the VV anastomosis	Total diameter of VV anastomosis. [18]
	Arteriovenous (AV) anastomoses	Number of arterio-venous anastomoses. [18]
	Venoarterial (VA) anastomoses	Number of venous-arterial anastomoses [18]
	Total anastomoses	Total number of anastomoses, by adding up all AA-, VV-, AV- and VA-anastomoses. [18]
	Cord insertion	The cord insertion [19]:<br>• Central<br>• Marginal (within 1 cm of placental margin)<br>• Velamentous
	Placental share smaller twin	Fetal territory of the smaller twin which should be measured by following the margins demarcated by the presence of color-specific dye. [19]
	Placental share discordance	Placental share discordancy is calculated as larger placental share−smaller placental share/larger placental share×100). [18]
	Placental territory ratio	Larger placental share divided by the smaller placental share[18]

TYPE	STUDY PARAMETER	ASSESSMENT TOOL/DEFINITION (4/4)
**Secondary Study characteristics (Neonatal characteristics)**	Sex	Male or female
	Birthweight	Weight directly measured after birth.
	Gestational age at birth	Gestational age at which the surviving cotwin was born.
	APGAR score	APGAR score at 5 and 10- minutes. [26]
	Perinatal asphyxia	At least one of the following criteria:<br>• Need for resuscitation<br>• Cord or early neonatal blood pH <7.0, base excess <−16 mmol/L, or lactate >10.0 mmol/L within 1 hour after birth<br>• Apgar score ≤5 after 5 minutes<br>• Spontaneous breathing > 5 minutes<br>• Mechanical ventilation for ≥10 minutes after birth
	Neonatal death	Neonatal death defined as death within 28 days after birth. [18]
	Age at neonatal death	Age in postnatal days at neonatal death
	Cause of neonatal death	Underlying disease which is the cause of death
	Respiratory distress syndrome (RDS)	Requiring mechanical ventilation and/or surfactant.
	Persistent pulmonary hypertension (PPHN)	Persistent pulmonary hypertension treated with inhaled nitro oxygen.
	Patent ductus arteriosus (PDA)	Requiring medical therapy or surgical closure.
	Necrotizing enterocolitis (NEC)	≥ grade 2 [20]
	Proven neonatal sepsis	Clinically ill neonate with a positive blood culture
	Retinopathy of prematurity (ROP)	Staged according to ICROP [21]
	Bronchopulmonary dysplasia (BPD)asphu	Need for supplemental oxygen for at least 28 days after birth, severity graded according to the respiratory support required at 36 postmenstrual weeks [22]
	Hemoglobin at birth	Hemoglobin level at birth in g/dl
	Use of inotropes	Use of intravenous inotropics; dopamine, dobutamine, adrenaline, noradrenaline
	Blood transfusion neonatal period	Any blood transfusion given in the first 28 days after birth
	Neurodevelopmental outcome	**Cognitive and motor test scores:**<br>Assessed using standardized psychometric age-appropriate tests:<br>• 2-3 yrs. ~ Bayley Scales of Infant and Toddler Development 3rd edition (Bayley-III) [27]<br>• 4-6 yrs. ~ Wechsler Preschool and Primary Scale of Intelligence 3rd edition (WPPSI-III) [28]<br>• 7-16 yrs. ~ Wechsler Intelligence Scale for Children 5th edition (WISC-V) [29]<br><br>**Cerebral palsy (CP):**<br>Assessed via a neurological examination according to Touwen in children aged 2-18 years.[30] Severity classified according to the GMFCS.[31]
	Ages and stages questionnaire (ASQ)	Screening tool measures development in 5 domains.[15]
	Parent Report of Children's Abilities-Revised (PARCA)	Assessment of cognitive and language development at age 2 years. [32]
	Mild visual impairment	Treatment by an ophthalmologist, strabismus, or a correction of max. +/- 3.

Severe visual impairment	Blindness or partially sighted
Mild hearing impairment	Hearing loss up to 30 dB.
Severe hearing impairment	Bilateral deafness, treatment in an audiological center, neurosensory hearing loss or hearing loss requiring amplification.
Mild neurodevelopmental impairment	Defined as at least one of the following:<br>• Mild cognitive/motor delay<br>• Mild visual impairment<br>• Mild hearing impairment<br>• CP stage 1
Severe neurodevelopmental impairment	Defined as at least one of the following:<br>• Severe cognitive/motor delay<br>• Severe visual impairment<br>• Severe hearing impairment<br>• CP ≥ stage 2

STATISTICAL ANALYSIS

Statistical data were analysed using SPSS version 28.0 (IBM, Armonk, NY, USA). Categorical data are presented as numbers and percentages; continuous variables are presented as mean with standard deviation for normally distributed variables, and medians with interquartile range for variables that are not normally distributed. Statistical analysis was performed using the t-test and Mann–Whitney U-test for continuous variables, whereas the chi-squared test and Fisher's exact test were used for categorical variables. The risk factors possibly contributing to severe brain injury were studied using a univariate regression model. Results are expressed as odds ratios (OR) with 95% confidence intervals (CI, and statistical significance was set at $p < 0.05$.

ETHICAL CONSIDERATIONS

Regulation statement
This study was conducted according to the principles of the Declaration of Helsinki (64th WMA General Assembly, October 2013) and the General Data Protection Regulation (GDPR).

Consent
Local investigators will submit the study protocol to their institutional review board for ethical approval (Figure 1). Our application will be handled according to the local and/or national rules and procedures applied in participating foreign centers. Each site will be responsible for obtaining ethical consent if required by their institution in collaboration with the study coordinator.

The regular consent procedure required the consent of the infants' parents before participation in the study. Each site will be responsible for obtaining ethical approval if required by their institution in collaboration with the study coordinator. We will prioritize centers where a waiver of consent has been granted over centers where opt-out or regular consent is required to reduce consent-related bias.

LUMC
June 2017 parents are informed in writing concerning the use of medical records for retrospective studies by providing a brochure. This brochure is standardly given during admittance to the neonatology ward in the LUMC. Coded data from infants admitted to the NICU are automatically included in databases for medical research except if parents have objections (opt-out procedure). If parents object the use of medical records for retrospective studies, notes will be made in the medical record and their infant(s) will not be included in retrospective studies.

The brochure was less consistently given to parents before June 2017. Therefore, we cannot assure all parents were informed with the opt-out procedure. However, patients of the department of Neonatology are a high-risk population for severe morbidity and mortality. Asking parents for consent to use data regarding their infant can be a huge psychological burden and is considered to be inappropriate. Because the collected data will be coded when stored, it cannot be traced back to individual patients without considerable effort. Participating in this study has no consequences for the patients or parents. Furthermore, data is collected for the purpose of improving neonatal care and this study may benefit prematurely born infants in the future. Also, as data starting from January 2000 will be collected it is likely that the majority of addresses and contact information is not up-to-date, and thus approaching parents individually for consent would be a considerable effort and is deemed unfeasible. These advantages are considered to outweigh the considerable effort of approaching all parents individually for consent.

Following the loss of one fetus in a twin pregnancy, parents often experience considerable stress during the initial phase and can feel overwhelmed by the abundance of information provided. The single survivors have an increased risk of severe morbidity and mortality. Requesting parental consent to utilize data regarding their infant in such circumstances can impose a significant psychological burden and is deemed inappropriate.

ADMINISTRATIVE ASPECTS, MONITORING AND PUBLICATION

Handling and storage of data and documents

Data will be collected, stored, and processed in accordance with the ICH E6 Good Clinical Practice (GCP)- Guidelines and the General Data Protection Regulation (GDPR). Electronic records will be held on a secure network at the participating centers. The data will be pseudonymized and will be uploaded to a Castor Database. To access this database the local investigator requires user ID and password to get access. Cases enrolled in the CITRUS study will be automatically assigned to a study number to encode all data directly after they are included in the study. The anonymization key will be safeguarded and kept by the local investigator of each participating center. Only the coordinating investigator and principal investigator will have access to the Castor database for analysis. The Regulations between the participating centers and the initiating center about the intended use, confidentiality, security and sharing of the data, will be arranged in a data sharing agreement (DSA). The DSA will be signed by each participating center separately.

Study data will be entered into Castor, a certified electronic database for collection and analysis. This database will be managed by the coordinating investigator and principal investigator from the LUMC. Castor complies with all applicable laws and regulations, including ICH E6 Good Clinical Practice (GCP) and by using Castor, researchers are enabled to comply with these laws and regulations (www.castoredc.com). Coded data will be stored in a Leiden University Medical Center ProMISe DataSafe. ProMISe meets the requirements for data safety and privacy set by international law. The ProMISe system facilitates the availability, integrity, and confidentiality of study data, according to the security conditions demanded by GCP. All data is coded and stored on Datasafes, that are exclusively accessible for the involved researchers. The ProMISe The dataset automatically generates backups twice a day. Data will be stored for the length of the study and 15 years afterwards.

Monitoring and Quality Assurance

The coordinating investigator and principal investigator will monitor the incoming data during the data collection period for inconsistencies, data entry errors, and missing data. Data acquired by this study will be available for inspection by the national and local health authorities, if applicable, upon request. Given the neglectable risk associated with this observational study, no data safety monitoring board will be installed.

Amendments

Not applicable in the LUMC since amendments to the protocol of non-WMO studies do not have to be submitted to the METC.

(Prematurely) End of study

Did not receive sufficient data

Publication policy

The results from the CITRUS Study will be analyzed and published as soon as possible in open access peer-reviewed international scientific journals and presented at scientific meetings unless the study was terminated prematurely and did not yield sufficient data for a publication. The responsibility for presentations and/or publications belongs to the principal investigators. No restriction regarding the public disclosure and publication of the research data have been or will be made by the funding agencies. All papers written as a part of this study will list the funding agencies. The final publication of the study results will be written by the principal investigators and the co- investigators. Local investigators will be named as co-authors on any publications resulting from this study, local coordinating investigators will be named under the CITRUS- group authorship (max. 3 investigators per participating center). The order of coauthors in the manuscript will be determined according to the number of patients each center contributed, listed from the most to the fewest cases provided. Other contributors to the study will be named as collaborators in a separate list added to the publication. A draft manuscript will be submitted for review to all co-authors. Results will also be published in a PhD-thesis.

REFERENCES

1. Lopriore E, Lewi L, Khalil A. Monochorionic Twins: A Delicate Balance. *J Clin Med*. Oct 17 2019;8(10)doi:10.3390/jcm8101711
2. Groene SG, Tollenaar LSA, Oepkes D, Lopriore E, van Klink JMM. The Impact of Selective Fetal Growth Restriction or Birth Weight Discordance on Long-Term Neurodevelopment in Monochorionic Twins: A Systematic Literature Review. *J Clin Med*. Jun 28 2019;8(7)doi:10.3390/jcm8070944
3. Tollenaar LSA, Slaghekke F, van Klink JMM, et al. Twin-Twin Transfusion Syndrome with Anemia-Polycythemia: Prevalence, Characteristics, and Outcome. *J Clin Med*. Jul 30 2019;8(8)doi:10.3390/jcm8081129
4. Groene SG, Tollenaar LSA, van Klink JMM, et al. Twin-Twin Transfusion Syndrome with and without Selective Fetal Growth Restriction Prior to Fetoscopic Laser Surgery: Short and Long-Term Outcome. *J Clin Med*. Jul 3 2019;8(7)doi:10.3390/jcm8070969
5. Griffiths PD, Sharrack S, Chan KL, Bamfo J, Williams F, Kilby MD. Fetal brain injury in survivors of twin pregnancies complicated by demise of one twin as assessed by in utero MR imaging. *Prenat Diagn*. Jun 2015;35(6):583–91. doi:10.1002/pd.4577
6. Moradi B, Badraqe N, Rahimi Sharbaf F, et al. Early detection of ischemic brain injuries by diffusion-weighted imaging after radiofrequency ablation for fetal reduction in monochorionic pregnancies. *J Clin Ultrasound*. Nov 2022;50(9):1288–1296. doi:10.1002/jcu.23237
7. O'Donoghue K, Rutherford MA, Engineer N, Wimalasundera RC, Cowan FM, Fisk NM. Transfusional fetal complications after single intrauterine death in monochorionic multiple pregnancy are reduced but not prevented by vascular occlusion. *BJOG*. May 2009;116(6):804–12. doi:10.1111/j.1471-0528.2009.02128.x
8. Shinar S, Harris K, Van Mieghem T, et al. Early imaging predictors of fetal cerebral ischemic injury in monochorionic twin pregnancy complicated by spontaneous single intrauterine death. *Ultrasound Obstet Gynecol*. Apr 2022;59(4):497–505. doi:10.1002/uog.24844
9. van Klink JM, van Steenis A, Steggerda SJ, et al. Single fetal demise in monochorionic pregnancies: incidence and patterns of cerebral injury. *Ultrasound Obstet Gynecol*. Mar 2015;45(3):294–300. doi:10.1002/uog.14722
10. Rondagh M, Lopriore E, de Vries LS, et al. The Current Knowledge of Cerebral Magnetic Resonance Imaging in Monochorionic Twins: A Systematic Review of the Last 20 Years. *J Clin Med*. Nov 21 2023;12(23)doi:10.3390/jcm12237211
11. Conte G, Righini A, Griffiths PD, et al. Brain-injured Survivors of Monochorionic Twin Pregnancies Complicated by Single Intrauterine Death: MR Findings in a Multicenter Study. *Radiology*. Aug 2018;288(2):582–590. doi:10.1148/radiol.2018171267
12. Spruijt MS, van Klink JMM, de Vries LS, et al. Fetal and neonatal neuroimaging in twin-twin transfusion syndrome. *Ultrasound Obstet Gynecol*. Jan 12 2024;doi:10.1002/uog.27583
13. de Vries LS, Eken P, Dubowitz LM. The spectrum of leukomalacia using cranial ultrasound. *Behav Brain Res*. Jul 31 1992;49(1):1–6. doi:10.1016/s0166-4328(05)80189-5
14. Volpe JJ. Confusions in Nomenclature: "Periventricular Leukomalacia" and "White Matter Injury"-Identical, Distinct, or Overlapping? *Pediatr Neurol*. Aug 2017;73:3–6. doi:10.1016/j.pediatrneurol.2017.05.013
15. Muthusamy S, Wagh D, Tan J, Bulsara M, Rao S. Utility of the Ages and Stages Questionnaire to Identify Developmental Delay in Children Aged 12 to 60 Months: A Systematic Review and Meta-analysis. *JAMA Pediatr*. Oct 1 2022;176(10):980–989. doi:10.1001/jamapediatrics.2022.3079
16. Gratacos E, Lewi L, Munoz B, et al. A classification system for selective intrauterine growth restriction in monochorionic pregnancies according to umbilical artery Doppler flow in the smaller twin. *Ultrasound Obstet Gynecol*. Jul 2007;30(1):28–34. doi:10.1002/uog.4046
17. Quintero RA, Morales WJ, Allen MH, Bornick PW, Johnson PK, Kruger M. Staging of twin-twin transfusion syndrome. *J Perinatol*. Dec 1999;19(8 Pt 1):550–5.

doi:10.1038/sj.jp.7200292
18. Groene SG, Openshaw KM, Jansén-Storbacka LR, et al. Impact of placental sharing and large bidirectional anastomoses on birthweight discordance in monochorionic twins: a retrospective cohort study in 449 cases. *Am J Obstet Gynecol*. Nov 2022;227(5):755.e1–755.e10. doi:10.1016/j.ajog.2022.05.059
19. Lopriore E, Sueters M, Middeldorp JM, Oepkes D, Walther FJ, Vandenbussche FP. Velamentous cord insertion and unequal placental territories in monochorionic twins with and without twin-to-twin-transfusion syndrome. *Am J Obstet Gynecol*. Feb 2007;196(2):159.e1–5. doi:10.1016/j.ajog.2006.10.865
20. Bell MJ, Ternberg JL, Feigin RD, et al. Neonatal necrotizing enterocolitis. Therapeutic decisions based upon clinical staging. *Ann Surg*. Jan 1978;187(1):1–7. doi:10.1097/00000658-197801000-00001
21. An international classification of retinopathy of prematurity. The Committee for the Classification of Retinopathy of Prematurity. *Arch Ophthalmol*. Aug 1984;102(8):1130–4. doi:10.1001/archopht.1984.01040030908011
22. Jobe AH, Bancalari E. Bronchopulmonary dysplasia. *Am J Respir Crit Care Med*. Jun 2001;163(7):1723–9. doi:10.1164/ajrccm.163.7.2011060
23. Khalil A, Beune I, Hecher K, et al. Consensus definition and essential reporting parameters of selective fetal growth restriction in twin pregnancy: a Delphi procedure. *Ultrasound Obstet Gynecol*. Jan 2019;53(1):47–54. doi:10.1002/uog.19013
24. Middeldorp JM, Lopriore E, Sueters M, et al. Twin-to-twin transfusion syndrome after 26 weeks of gestation: is there a role for fetoscopic laser surgery? *Bjog*. Jun 2007;114(6):694–8. doi:10.1111/j.1471-0528.2007.01337.x
25. Winer N, Caroit Y, Le Vaillant C, Philippe HJ. [Monoamniotic twins: diagnosis and management]. *J Gynecol Obstet Biol Reprod (Paris)*. Dec 2009;38(8 Suppl):S85–9. Grossesse monochoriale monoamniotique: diagnostic et prise en charge. doi:10.1016/s0368-2315(09)73564-1
26. Simon LV, Hashmi MF, Bragg BN. APGAR Score. *StatPearls*. StatPearls Publishing Copyright © 2023, StatPearls Publishing LLC.; 2023.
27. Bayley N. Bayley scales of infant and toddler development–Third edition. *Pearson Education Inc*. 2006;
28. Wechsler D. Wechsler Preschool and Primary Scale of Intelligence - Third Edition. *The Psychological corporation*. 2002;
29. Wechsler D. Wechsler Intelligence Scale for Children (5th ed.). *NCS Pearson*. 2014;
30. Touwen BC, Hempel MS, Westra LC. The development of crawling between 18 months and four years. *Dev Med Child Neurol*. May 1992;34(5):410–6. doi:10.1111/j.1469-8749.1992.tb11453.x
31. Palisano R, Rosenbaum P, Walter S, Russell D, Wood E, Galuppi B. Development and reliability of a system to classify gross motor function in children with cerebral palsy. *Dev Med Child Neurol*. Apr 1997;39(4):214–23. doi:10.1111/j.1469-8749.1997.tb07414.x
32. Johnson S, Bountziouka V, Brocklehurst P, et al. Standardisation of the Parent Report of Children's Abilities-Revised (PARCA-R): a norm-referenced assessment of cognitive and language development at age 2 years. *Lancet Child Adolesc Health*. Oct 2019;3(10):705–712. doi:10.1016/s2352-4642(19)30189-0
