# Supplementary material for: Brain injury after single fetal demise in monochorionic twin pregnancies: an international multicentre retrospective cohort study
Source: eClinicalMedicine. 2026 Oct 28;101:104290. doi: 10.1016/j.eclinm.2026.104290 (PMC13735160; doi:10.1016/j.eclinm.2026.104290)
Supplement: Supplementary file 2 [file mmc2.docx]

**CITRUS Study Group members**

| **First names** | **Surnames** |
| --- | --- |
| Ana | Alarcón |
| Mar | Bennasar |
| Giana | Izzo |
| Daniela | Longo |
| Marcelo | Pietrani |
| Borna | Poljak |
| Sara | Savelli |
| Irene | Spektor |
| Federica | Torracca |
